# Detailed Phenotypic and Molecular Analyses of Genetically Modified Mice Generated by CRISPR-Cas9-Mediated Editing

**DOI:** 10.1371/journal.pone.0116484

**Published:** 2015-01-14

**Authors:** Bijal A. Parikh, Diana L. Beckman, Swapneel J. Patel, J. Michael White, Wayne M. Yokoyama

**Affiliations:** 1 Department of Pathology and Immunology, Washington University School of Medicine, St. Louis, MO, 63110, United States of America; 2 Division of Rheumatology, Department of Medicine, Washington University School of Medicine, St. Louis, MO, 63110, United States of America; 3 Howard Hughes Medical Institute, Washington University School of Medicine, St. Louis, MO, 63110, United States of America; University of Florida, USA

## Abstract

The bacterial CRISPR-Cas9 system has been adapted for use as a genome editing tool. While several recent reports have indicated that successful genome editing of mice can be achieved, detailed phenotypic and molecular analyses of the mutant animals are limited. Following pronuclear micro-injection of fertilized eggs with either wild-type Cas9 or the nickase mutant (D10A) and single or paired guide RNA (sgRNA) for targeting of the *tyrosinase* (*Tyr*) gene, we assessed genome editing in mice using rapid phenotypic readouts (eye and coat color). Mutant mice with insertions or deletions (indels) in *Tyr* were efficiently generated without detectable off-target cleavage events. Gene correction of a single nucleotide by homologous recombination (HR) could only occur when the sgRNA recognition sites in the donor DNA were modified. Gene repair did not occur if the donor DNA was not modified because Cas9 catalytic activity was completely inhibited. Our results indicate that allelic mosaicism can occur following -Cas9-mediated editing in mice and appears to correlate with sgRNA cleavage efficiency at the single-cell stage. We also show that larger than expected deletions may be overlooked based on the screening strategy employed. An unbiased analysis of all the deleted nucleotides in our experiments revealed that the highest frequencies of nucleotide deletions were clustered around the predicted Cas9 cleavage sites, with slightly broader distributions than expected. Finally, additional analysis of founder mice and their offspring indicate that their general health, fertility, and the transmission of genetic changes were not compromised. These results provide the foundation to interpret and predict the diverse outcomes following CRISPR-Cas9-mediated genome editing experiments in mice.

## Introduction

Genome editing is a powerful approach used to analyze biological functions in whole organisms as well as at the single cell level. In mice, gene targeting has traditionally been accomplished in embryonic stem (ES) cells via homologous recombination (HR) of donor DNA in a process that utilizes the host DNA repair machinery without deliberate induction of a site-specific double strand break (DSB). This approach has been used to modify a variety of sequences from multi-gene arrays to single nucleotide mutations (SNM). This method, however, is highly inefficient and requires the use of a selectable marker to identify recombinants [1]. Moreover, the time from initial planning to generation of a homozygous knockout mouse can often take more than a year and require dedicated personnel; even then, targeted mutations may not be transmitted in the germline [2]. Consequently, many studies of mutated genes come from analysis of single founder lines.

Improved methods for genome editing have recently been developed whereby DNA is cleaved at precise locations. Zinc finger nucleases (ZFNs) and transcription activator-like effector nucleases (TALENs) are proteins which can be modified to cleave specific DNA sequences [3]. Although modular components can be customized for the desired DNA specificity, time is still required for optimization and testing of the modified nucleases to validate specificity. The newest approach for genome editing is based on the bacterial clustered, regularly interspaced, short palindromic repeats (CRISPR) defense system and is dependent on nucleic acid homology for site-specific DNA cleavage [4]. This method requires the endonuclease Cas9, a single guide RNA (sgRNA) with sequences that direct Cas9 to the genetic locus of interest, and a target site in the genome defined by 20 nucleotides (nt) of any base composition followed by an NGG protospacer adjacent motif (PAM) [5, 6]. By simply changing the sgRNA, CRISPR-Cas9-based approaches have been shown to be effective in generating genetic changes in a variety of mammalian cell lines [5]. Additionally, many eukaryotic model systems have also been successfully modified with CRISPR-Cas9, including *C. elegans* [7], zebrafish [8], and drosophila [9].

All three of these site-specific nucleases induce DSBs which are repaired by either the error-prone non-homologous end-joining (NHEJ) or the highly accurate HR pathways [10, 11]. NHEJ appears to be the default pathway for DSB DNA repair and its rapid resection and annealing of the DNA ends can result in no change, deletion, or insertion (indels) of a 1–30 nt at the repaired DSB site [12]. NHEJ can proceed through either the classical or alternative microhomology-mediated end-joining (MMEJ) repair mechanism, dependent on cell type and species. MMEJ frequently involves larger deletions (over several hundred nt) than observed with classical NHEJ [13]. Since classical NHEJ is the predominant form of DSB repair, small indels are the expected result of most DSB. In contrast, HR results in a highly accurate repair of the DSB with a DNA template required to provide “donor” sequences [14]. Several distinct mechanisms have been described for resolution of DNA ends via HR, including synthesis-dependent strand annealing (SDSA), double Holliday junction resolution, and single-strand annealing (SSA) [15]. Similar to MMEJ, SSA also involves a large DNA deletions over several hundred nt [13]. Thus, site-specific induction of DSB by nucleases facilitates generation of mutant alleles and HR.

Since CRISPR-Cas9-mediated cleavage can occur with high efficiency, researchers have taken advantage of HR following DSB to introduce foreign DNA into the mouse genome by pronuclear injection of CRISPR components into fertilized eggs [16]. The donor DNA template for repair via HR can be either a circular plasmid or ssDNA. Circular DNA allows for much larger insertions of foreign genetic elements, but it can be recognized and cleaved by Cas9 both before and after genomic incorporation. In addition to the limitation of Cas9 recognition, linear dsDNA may inadvertently result in random transgene insertion and concatemerization independent of the generation of DSBs [16]. ssDNA donor templates have the advantage of not being recognized by Cas9 as a target until it is incorporated in the genome, but these templates are limited by both the size of the desired insert and the homology arms.

Nearly all published studies have emphasized that CRISPR-Cas9 approaches can be successful for targeting specific murine genes of interest [17–27]. Other than targeting success, few data are available on the molecular outcomes of these approaches, adding to the difficulties in extrapolating these data to each other due to the different gene targets and guide RNAs. Moreover, pronuclear injection to induce genome editing, for example, has the potential to result in genetic mosaicism when more than two alleles are identified in founder animals. This outcome has been reported for both ZFN [28] and CRISPR-Cas9-mediated editing [25]. Mosaicism can confound phenotypic correlations in founder mice and influence the transmission of selected alleles to progeny, yet little is known about its frequency. Thus, detailed outcomes analyses from targeting of a single gene will be helpful in understanding the limitations and challenges of CRISPR-Cas9-mediated genome editing while also illuminating principles for analyzing future experiments.

An in-depth assessment of CRISPR-Cas9-mediated genome editing of the mouse germline would ideally employ both phenotypic and genotypic analyses. The phenotypic analysis itself would best be done on a gene that is not required for viability of the mouse and can be assessed during embryo development or soon after birth. The tyrosinase (*Tyr*) gene on chromosome 7, which is responsible for both black coat color and eye pigmentation in wild-type (WT) C57BL/6 (B6) mice [29], fulfills these criteria. The naturally occurring albino mutation within exon 1 of *Tyr* at nt 230 (numbering relative to the ATG translational start site) results in a change from leucine to arginine at amino acid 77. As an autosomal recessive trait, the absence of both WT copies of *Tyr* in the commonly used Albino B6 (AB6) strain results in a mouse with pink eyes at birth and white fur soon thereafter, whereas heterozygous mice, which have black eyes and fur, cannot be distinguished phenotypically from homozygous WT mice. A benefit of the eye color phenotype is that even still-born or non-viable pups can be analyzed for successful genome editing independent of sequencing. While *Tyr* disruption can be determined in wild-type or heterozygous (AB6xB6) F_1_ hybrid mice, gene correction via HR can be monitored in AB6 mice because only one corrected allele is needed to achieve a complete reversal of the pigmentation phenotype. Furthermore, mosaicism can easily be determined based on the distribution of the pigmentation. Thus, targeting of *Tyr* should provide a rapid phenotypic assessment of successful (or not) CRISPR-Cas9-mediated gene targeting in mice, complementing genotypic analysis.

We report a detailed analysis of *in vivo* CRISPR-Cas9-mediated *Tyr* gene editing. A combination of phenotypic and genotypic analyses allowed us to reveal the importance of donor DNA modification for successful HR and retention of Cas9 endonuclease activity, the frequency and molecular basis for mosaicism, the generation of very large deletions, and the lack of detectable off-target cleavage events. Additionally, compilation of our numerous gene modification experiments at a specific site in the mouse genome identified the site of Cas9 cleavage and demonstrated the influence of the NHEJ repair process on the diversity of observed genetic changes.

## Results

### Rapid phenotypic analysis of CRISPR-Cas9-mediated genome-editing

**Figure 1:**
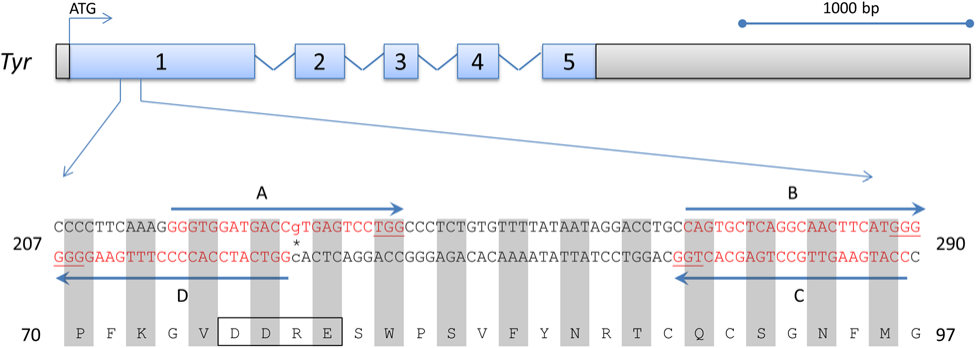
Tyrosinase gene and sgRNA placement. A representation of the WT B6 *Tyr* (NM_011661.4) coding sequence with the five exons (numbered) and flanking untranslated regions in gray. The region of interest for genome editing is enlarged. The SNM resulting in an eye and coat color change is indicated (*) and corresponds to nt 230 with reference to the translation start site. The amino acids encoded are shown below the DNA sequence and the critical “DDRE” motif for *Tyr* function is boxed. Four sgRNAs were designed flanking or including this site with indicated orientations (guides A, B, C, and D). The sgRNA binding sites on the homologous DNA are indicated in red, with the PAM sites underlined.

**Figure 2:**
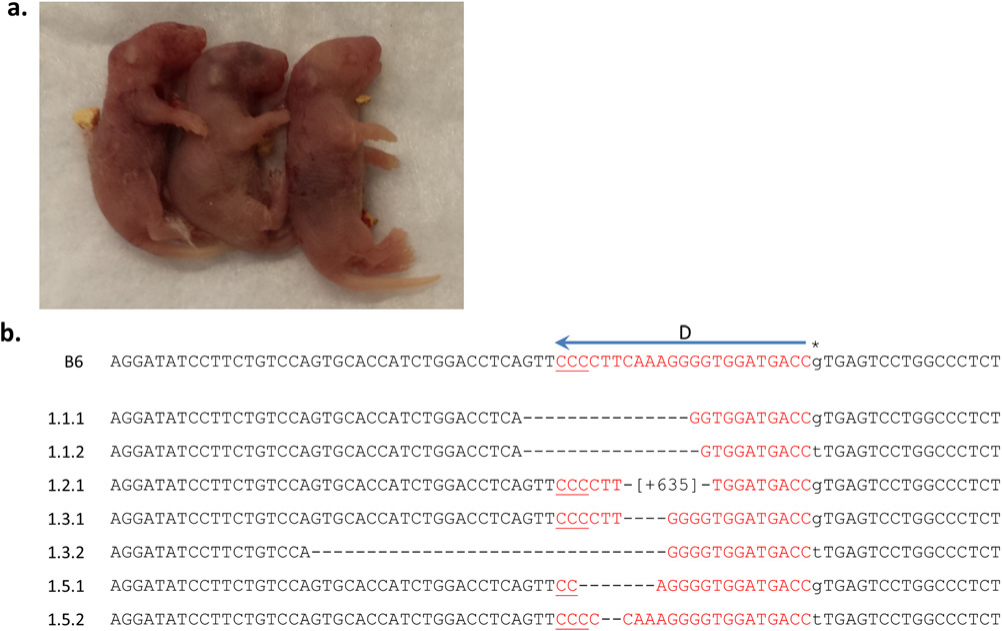
Successful genome editing of *Tyr* is evident on the day of birth and confirmed by DNA sequencing. **(a)** Phenotypic results of guide B editing of B6xAB6 fertilized eggs (Experiment 1) resulted in loss of eye pigmentation in two of three pups photographed on day 1 after birth, whereas one pups appeared unaltered (middle pup). Since sequencing analysis was not pursued in these pups, a disruptive indel on the AB6 allele and/or an in-frame indel on the B6 allele could also result in the same phenotypic finding. The mice in Experiment 1 did not survive because the cage housing the pups from guide B was flooded while pups from guide D were cannibalized by the mother. **(b)** Sequencing results of guide D editing from Experiment 1 are shown here. The *Tyr* alleles from guide B targeting were not analyzed. The B6 allele is indicated with the position of guide D as in Fig. 1. Mice are numbered with the following convention: (experiment#.mouse#.allele#). Mouse 1.1 (in-frame deletion on the B6 allele) and mouse 1.4 (no indels observed) had black eyes while the remaining mice had pink eyes at birth. Sequencing results from unaltered *Tyr* alleles are not shown. Deleted nt (-). Inserted nt in brackets.

Target regions for *Tyr* gene disruption by CRISPR-Cas9-mediated cleavage were identified as sequences of 23 nt having the canonical “NGG” PAM and a unique 5′ sequence unrelated to other genomic sequences (off-target sites). We designed four guides (A-D) that targeted opposite DNA strands in exon 1 of the *Tyr* locus, flanking nt 230 (Fig. 1), and screened them for minimal homology to potential off-target sites. While no perfect match sequence in the genome existed other than the intended sites within the *Tyr* gene, there was the possibility of homology if several mismatched nt were allowed. Using iterative BLAST searches with each of the four guide sequences, we failed to identify such homologous stretches of DNA comprised of less than three mismatches with the 20-nt sgRNA recognition sequence prior to the required “NGG” at additional genomic sites. We later verified this search with additional web-based algorithms during our off-target analyses (see below). We individually introduced each of these four sgRNAs along with WT Cas9 mRNA via pronuclear injection in a fertilized B6xAB6 ovum. Live births did not result from the injection of two sgRNAs (A and C; see Table 1, Experiment 1). In fact, none of the mice (targeted with any sgRNA) in Experiment 1 survived beyond several days. While this might be explained by sgRNA toxicity due to off-target effects, this could equally be explained by some technical factor outside of CRISPR-Cas9 toxicity such as handling of micro-injected eggs prior to implantation or cannabilization of normal pups by primigravid mothers. Nevertheless, as no DNA or phenotypic information was obtained following injection of guides A and C, we used them only once and did not utilize them in future experimental attempts which were designed to further understand successful CRISPR-Cas9 cleavage. By contrast, the other two sgRNAs (B and D) generated a majority of pups which showed abrogation of *Tyr* function (Tables 1, 2). Specifically, five of the eight pups analyzed had lost eye pigmentation (3 pups were photographed, Fig. 2a). Sequencing of the region targeted by guide D revealed that four of five pups had one or both alleles altered (Fig. 2b) with the majority resulting in deleterious mutations, such as out-of-frame mutations and premature stop codons (not shown). Interestingly, in the B6 allele of mouse 1.1 (1.1.1 in Fig. 2b), the deletion of 15 nt resulted in an in-frame mutation that preserved the critical amino acid residues required for *Tyr* function, DDRE. As a result, even though deletions were generated on both alleles, the eye color did not change, giving an underestimate of true targeting frequency at the genotype level. While most alterations were deletions, at least one allele (1.2.1) contained a 635 nt insertion. This insertion contained no obvious homology to *Tyr* and mapped to a region on chromosome 6 (data not shown). The alternate allele from this mouse could not be amplified for sequence analysis, suggesting an even larger deletion or rearrangement (see below). Thus, even though we directly used sgRNAs without *in vitro* testing, we were able to successfully target *Tyr* with efficiencies of greater than 60%.

**Table 1:** Tyrosinase genome editing experiments via CRISPR-Cas9.

**Experiment #**	**Host**	**CRISPR guide(s)**	**Cas9**	**Guide conc. (ng/µl)**	**Cas9 conc. (ng/µl)**	**Donor**	**Donor conc. (ng/µl)**	**# pups born**	**Days of Injection**	**# pups weaned (dead)**	**# pups w/color change**	**# pups w/indels**	**Frequency both alleles targeted**	**# pups with donor insertion**
1	AB6xB6 F1	A	WT	20	200			0	1					
		B	WT	20	200			3	1	0 (3)	2/3	n.d.	n.d.	
		C	WT	20	200			0	1					
		D	WT	20	200			5	1	0 (5)	3/5	4/5	4/4	
2	B6	B+D	D10A	20^a^	200			5	2	0 (5)	2/3^b^	1/2	1/2	
		B+D	WT	20^a^	200			8	2	4 (4)	3/8	6/8	4/6	
3	AB6	B+D	WT	20^a^	200	WT	10	5	1	5 (0)	0/5	0/5	n/a	
		B+D	D10A	20^a^	200	Mod	10	2	1	0 (2)	0/2	2/2	1/2	
		B+D	WT	20^a^	200	Mod	10	7	3	6 (1)	1/6^b^	3/6	2/3^e^	1
4	B6	B+D	D10A	20^a^	100	Mod	10	17	2	17 (0)	4/17^d^	6/17	4/6^e^	2
5	AB6	D	WT	10	100	WT	20	14	1	14 (0)	0/14	0/14	n/a	
		D	WT	10	100	WT	10	8	1	4 (4)	0/8	0/8	n/a	
		D	WT	10	100	Mod	20	8	2	4 (4)	0/8	4/8	2/4	
		D	WT	10	100	Mod	10	4^c^	2	0 (4)	0/4	3/4	2/3	

Individual experiments with the indicated number of injection attempts and resulting mice are shown. “Days of injection” refers to an independent reconstitution of RNA and/or DNA reagents, multiple rounds of micro-injection and subsequent implantation into several pseudo-pregnant recipients limited to a single day. All donor molecules were circular DNA and encoded the functional B6 allele. Donor refers to donor DNA with “WT” indicating that the donor DNA contained unmodified guide RNA recognition sites, whereas “Mod” indicates that the recognition sites were modified as described in the text.

^a^ Concentration of each guide (ng/ml)

^b^ Coat color and/or DNA not obtained from all pups

^c^ C-section was performed to deliver some of the pups

^d^ Phenotypic mosaic in one mouse

^e^ Genotypic mosaic in one or more mice

**Table 2:** Summary of all Cas9-mediated genome editing experiments.

**Results**		**Days of injection**	**Experiment #**	**# Mice positive**	**Total mice analyzed**	**Overall %**
Indel frequency						
Guide B	No donor present	1	1	2	3	66.7%
	Along with modified donor	not done				
Guide D	No donor present	1	1	4	5	80.0%
	Along with modified donor	4	5	7	12	58.3%
Guides B and D	No donor present (WT Cas9)	2	2	6	8	75.0%
	Along with modified donor (WT Cas9)	3	3	3	6	50.0%
	No donor present (D10A Cas9)	2	2	2	3	66.7%
	Along with modified donor (D10A Cas9)	3	3,4	8	19	42.1%
HR Repair						
Homologous recombination with guides B and D	Total	6	3,4	3	25	12.0%
	In mice with identified indels only	6	3,4	3	11	27.3%
	Along with modified donor and WT Cas9	3	3	1	6	16.7%
	Along with modified donor and D10A Cas9	3	4	2	19	10.5%
Homologous recombination with guide D	Along with modified donor and WT Cas9	4	5	0	12	0.0%
Donor DNA inhibition	Indels when co-injected with modified donor DNA	10	3,4,5	18	37	48.6%
	Indels when co-injected with native donor DNA	7	3,5	0	27	0.0%
Mosaicism and large deletions						
Mosaicism	*Genotypic:* Amongst all mice with indels	15	all	3	29	10.3%
	*Phenotypic:* Amongst adult mice with indels	15	all	1	18	5.6%
	*Phenotypic:* Amongst all mice	15	all	1	52	1.9%
Large (>600 nt) indels amongst all mice with indels	Total	15	all	6	29	20.7%
	Deletions	15	all	5	29	17.2%
	Insertions	15	all	1	29	3.4%

All of the data from mice analyzed across multiple injection days and experimental conditions were pooled to generate the comparisons depicted here. This table summarizes the primary data shown in the other Tables and Figures.

### Cleavage with paired sgRNAs and WT Cas9 or the Cas9 nickase mutant

**Figure 3:**
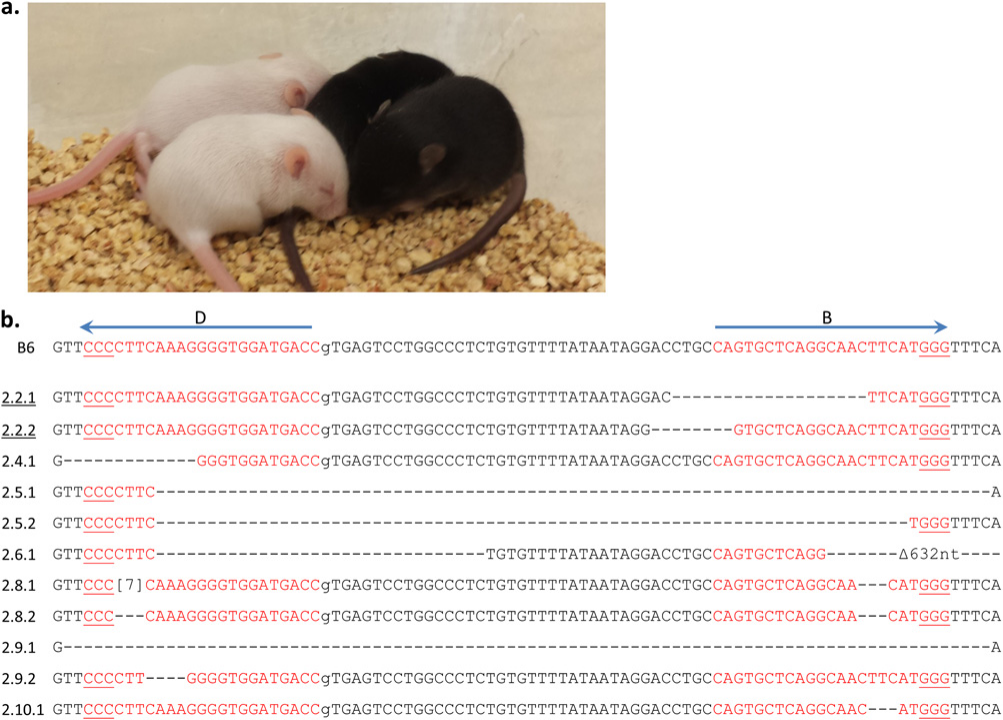
Paired sgRNAs result in gene disruption by phenotype and DNA sequencing. **(a)** Phenotypic results of guide B and D editing of B6xB6 fertilized eggs (Experiment 2) resulted in loss of black coat color in two of four pups photographed on day 14 after birth. The following mouse numbers and their phenotypes are shown: 2.3 (black), 2.4 (black), 2.5 (white), 2.6 (white). **(b)** Sequencing results of combined guide B and D editing are depicted. The B6 allele is indicated with the positions of guides B and D as in Fig. 1. Mice are numbered as in Fig. 2b. Sequencing results from unaltered *Tyr* alleles are not shown (2.4.2, 2.10.2). No PCR product was detected for allele 2.6.2; later it was confirmed to be a large 42 kb deletion (Fig. 8). Underlined alleles were from a D10A Cas9 injection. Deleted nt (-). Inserted nt in brackets.

Given the high efficiency of single guides alone, we next determined whether simultaneous injection of both guides would influence the observed cleavage frequency. A prior report of dual sgRNA targeting suggested that this approach resulted in a higher frequency of indels [30]. Furthermore, we reasoned that a high rate of cleavage would generate indels on both WT *Tyr* alleles in a single cell, resulting in an observable phenotype. Simultaneous introduction of guides B and D into a B6 zygote homozygous for the WT *Tyr* alleles resulted in five of eleven pups having a coat color change (Experiment 2, Table 1, Fig. 3a). The sequences of alleles from ten of these pups (one albino pup was not sequenced) confirmed the albino mutations and also showed three additional pups had heterozygous *Tyr* alterations (Table 1, Fig. 3b). Based on both phenotypic and sequence analysis, five pups were targeted on both alleles, three on only one allele, and three on neither allele.

Additionally, it appeared that the targeting efficiency at one site was not affected by targeting at the other. Even though both guides were injected, three of the alleles were targeted only with guide B, two only with guide D, and six with indels at both sites (Fig 3b). Only three of these paired deletions resulted in loss of the intervening sequence suggesting that the DSB does not need to occur synchronously. This finding implies that one DSB could be repaired prior to the initiation of the second break. Using the single guide D cleavage frequency of 80% for reference (Table 2), we found a similar rate of cleavage at this target site when multiplexed with guide B. Specifically, of the seven pups with identified indels in Experiment 2 (Table 2, Fig. 3b), only one failed to harbor an allele with cleavage at the guide D recognition site (mouse 2.2). Overall, these results suggest that both sgRNAs are acting equally and independently and that the success rate at either sgRNA recognition site was not markedly affected by multiplexing the sgRNAs.

**Figure 4:**
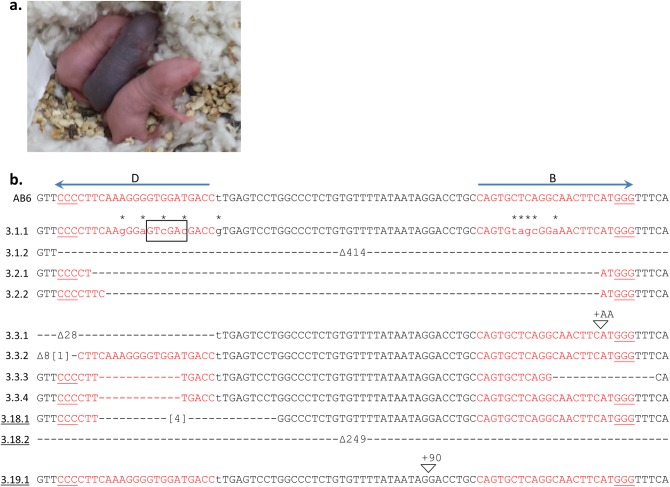
Successful gene repair of point mutation by phenotype and DNA sequencing. **(a)** Phenotypic results of guide B and D editing of AB6xAB6 fertilized eggs (Experiment 3) resulted in a gain of black coat color in one of three pups photographed on day 5 after birth. Mice 3.1 (black pup), 3.2, and 3.3 are shown. **(b)** The results of combined guide B and D editing in AB6 mice with a donor template are depicted. When a modified donor was used, the mutations are indicated by (*) above the lowercase base substitutions. Mice are numbered as in Fig. 2b. The AB6 allele is indicated with the positions of guides B and D as in Fig. 1. Sequencing results from the unaltered *Tyr* allele 3.19.2 is not shown. Mouse 3.3 is a genetic but not phenotypic mosaic. Underlined alleles were from a D10A Cas9 injection. Deleted nt (-). Inserted nt in brackets. The introduction of a novel *Sal*I restriction enzyme site is identified by a box for allele 3.1.1.

**Figure 5:**
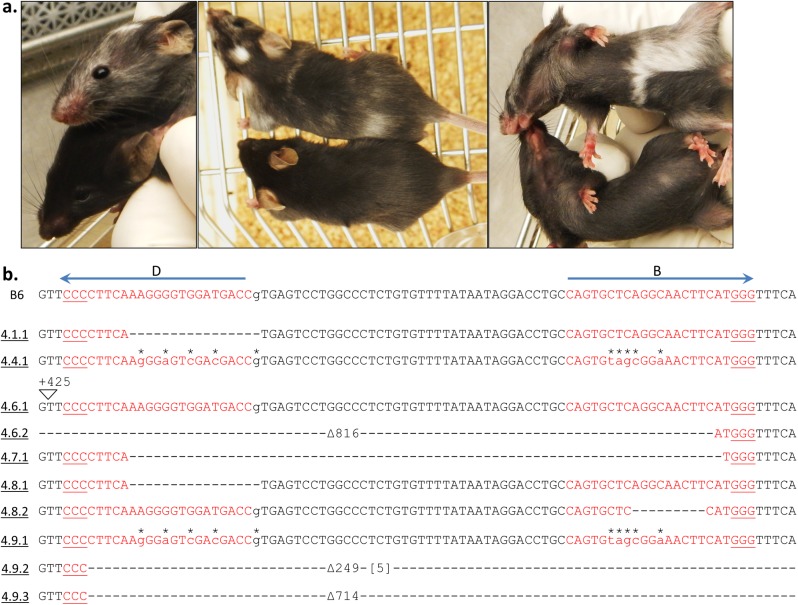
Mosaicism following CRISPR-Cas9-mediated genome-editing. **(a)** Mouse 4.9 is shown in three photographs (top) along with a WT B6 littermate (bottom) at four weeks of age. The patchy coat color distribution was evident several days after birth and has remained consistent throughout development. **(b)** The results of combined guide B and D editing in B6 mice with a donor template are depicted. The mutations present in the modified donor are indicated by (*) above the lowercase base substitutions. Mice are numbered as in Fig. 2b. The B6 allele is indicated with the positions of guides B and D as in Fig. 1. Sequencing results from unaltered *Tyr* alleles are not shown (4.1.2, 4.4.2, 4.8.3). No PCR product was detected for allele 4.7.2 suggesting a large indel. Deleted nt (-). Inserted nt in brackets.

The nickase mutant of Cas9 (D10A) also recognizes double-stranded DNA, but cleaves only the complementary strand [31]. D10A Cas9 can also generate a second single-strand break on the opposite strand when a second sgRNA target site is located nearby. A prior report of the optimal offset between targets revealed that excision of the DNA between the two single-strand cleavage sites rapidly decreased at distances greater than 100 nt and was critically dependent on the orientation of the two target PAM sites [32]. Fortuitously, guides B and D are oriented in opposite directions with an offset between the guide sites of 38 nt and 5′ overhang distance of 72 nt. As summarized in Table 2, we maintained a greater than 60% indel frequency when paired sgRNAs were co-injected with either WT or D10A Cas9. However, the possibility of the Cas9 nickase functioning in some capacity as a WT Cas9 nuclease with DSB has been reported, albeit at a very low frequency [33]. In fact, we have found in four of our D10A targeted mice, only one of the two guide sites contained deletions (mice 2.2, 3.18, 4.1 and 4.8; Figs. 3, 4, 5). Our findings support the possibility of residual double-strand DNA cleavage by D10A Cas9 as the region between the two guide sites was not consistently lost.

### Gene repair did not occur with donor DNA having intact guide recognition sites

Donor DNA for HR gene repair was generated with 800 nt WT *Tyr* homology arms (WT donor) on either side of the intended (AB6→B6) *Tyr* substitution at nt position 230 (Fig. 1, Materials and Methods). We co-injected this circular WT donor DNA molecule along with Cas9 mRNA and either one sgRNA (Experiment 3, Table 1) or two sgRNAs (Experiment 5, Table 1) into an AB6 host. Remarkably, in the 27 mice that were analyzed, we did not obtain any mice in which the donor DNA had been inserted at the *Tyr* locus (Table 2). One of these mice (5.3) appeared to have incorporated the WT donor DNA as a random transgene based on the following evidence: the coat color of the AB6 founder remained white; the donor DNA was amplified using vector specific primers in the founder; and offspring following mating to AB6 were all white (Table 3) indicating a non-functional insert. Even more striking was that none of the 27 mice following WT donor micro-injection demonstrated indel generation, even though we used the same guides previously shown to target *Tyr* with greater than 60% efficiency (Tables 1,2).

**Table 3:** Transmission and distribution of mutant alleles in F1 offspring.

**Mouse #**	**Strain of Fertilized Egg**	**Coat Color**	**Alleles**	**Litters (pups)**	**Color distribution**	**Number sequenced**	**Allele distribution:Allele (counts)**
2.3	B6	Black	Both WT	2 (7)	All	3	All WT
2.4	B6	Black	1 = Δ205–217	4 (31)	17/31	6	1 (3)
			2 = WT				2 (3)
2.5	B6	White	1 = Δ214–294	5 (26)	All	20	1 (11)
			2 = Δ214–286				2 (9)
2.6	B6	White	1 = Δ214–245; Δ279–910	4 (30)	All	18	1 (10)
			2 = Large Deletion				2 (8)
3.1	AB6	Black	1 = Gene correction	5 (22)	11/22	7	1 (4)
			2 = Δ207–620				2 (3)
3.2	AB6	White	1 = Δ212–295	2 (11)	All	11	1 (5)
			2 = Δ214–285				2 (6)
3.3	AB6	White	1 = Δ202–229; 285 [+AA]	2 (11)	All	11	1 (3)
			2 = Δ202–208; 208 [+T]				2 (1)
			3 = Δ213–224; Δ279–293				3 (3)
			4 = Δ213–224				4 (1)
			5 = WT AB6				5 (3)
4.4	B6	Black	1 = Gene Correction	1 (5)	All	5	1 (1)
			2 = WT B6				2 (4)
4.7	B6	White	1 = Δ215–286	1 (6)	All	6	1 (3)
			2 = Large deletion				2 (3)
4.8	B6	White	1 = Δ215–230	4 (28)	All	28	2 (14)
			2 = Δ276–284				4 (14)
			3 = WT B6				
			4 = Large deletion				
4.9	B6	80%	1 = Gene correction	1 (5)	4/5	5	1 (4)
			2 = Δ213–926				2 (1)
			3 = Δ210–399 [+5]				
5.1	AB6	White	1 = Δ202–220	2 (12)	All	12	1 (7)
			2 = Δ212–215				2 (5)
5.2	AB6	White	1 = Δ199–220	2 (16)	All	16	1 (4)
			2 = ins 212 [+A]				2 (4)
5.3	AB6	White	1 = WT AB6	2 (14)	All	14	1 (6)
			2 = B6 donor transgene				2 (8)

Mice that reached adulthood were crossed with AB6 mice to track genetic and phenotypic changes following germline transmission. Only mice that generated progeny are shown here. Coat color refers to the founder mouse whereas color distribution refers to the phenotype of the offspring. The nt deleted are indicated with reference to the ATG translational start site. Insertions at the indicated positions are depicted in brackets as either the number of nt inserted or their identity.

### Gene repair of a single nucleotide with WT or D10A Cas9

Based on the inability to generate indels when we co-injected Cas9, sgRNA and WT donor DNA, we hypothesized that the donor itself could be a substrate for Cas9-dependent cleavage and adversely affect HR gene repair. Although simply altering the GG dinucleotide of the PAM could be sufficient to test this idea, altering this motif in our *Tyr* donor DNA would unavoidably affect the coding potential. Instead we modified the sgRNA recognition sites by using alternate codons that maintained the translated protein sequence (Fig. 4b, Table 4, Materials and Methods). These modifications introduced four to five mismatches in the guide B and D sites and a novel *Sal*I restriction enzyme site to facilitate donor detection (Fig. 4b). Our initial results in AB6 mice with this modified donor template and WT Cas9 resulted in a pup that phenotypically exhibited the “repaired” B6 pigmentation (Fig. 4a). Although it was possible that this phenotype could have occurred by chance reversion of a single nt, our sequence analysis showed that this mouse (3.1) contained a “repaired” allele with the modified sequence from the donor DNA. Moreover, it also contained a deletion on the alternate allele (Fig. 4b) and we found indels in five of eight mice without a repaired *Tyr* allele (Experiment 3, Table 1). Thus, the CRISPR-Cas9 approach can generate both a gene knockin and a knockout in a single mouse that can serve to generate two independent founder lines.

**Table 4:** Primer Sequences.

**sgRNA primers**	**Sequence**
Guide A	5′-TTAATACGACTCACTATAGGGGTGGATGACCTTGAGTCCGTTTTAGAGCTAGAAATAGCAAG-3′
Guide B	5′-TTAATACGACTCACTATAGGCAGTGCTCAGGCAACTTCATGTTTTAGAGCTAGAAATAGCAAG-3′
Guide C	5′-TTAATACGACTCACTATAGGCCATGAAGTTGCCTGAGCACGTTTTAGAGCTAGAAATAGCAAG-3′
Guide D	5′-TTAATACGACTCACTATAGGGTCATCCACCCCTTTGAAGGTTTTAGAGCTAGAAATAGCAAG-3′
Universal sgRNA Reverse	5′-AAAAGCACCGACTCGGTGCC-3′
**Cas9 primers**	
px330 (WT) Forward	5′-TAATACGACTCACTATAGGGAGAATGGACTATAAGGACCACGAC-3′
px330 (WT) Reverse	5′-GCGAGCTCTAGGAATTCTTAC-3′
px335 (D10A) Forward	5′-TAATACGACTCACTATAGGGAGAATGTACCCATACGATGTTCCAG-3′
px335 (D10A) Reverse	5′-GCGAGCTCTAGGAATTCTTAG-3′
**Sequencing primers**	
Tyr Fwd 1	5′-CTCATTAACCTATTGGTGCAGATT-3′
Tyr Rev 1 (Product size = 989nt)	5′-GCATTAACATCTGTTAGTAAGGCA-3
Tyr Fwd 2	5′-CTAGAAACTTTATGCATTGAAGCAG-3′
Tyr Rev 2 (Product size = 1679nt with For 2; 1555nt with For 1)	5′-CAGTCCTTGTTTATAGCAGCTTAG-3′
Tyr Fwd 3	5′-GCCCTTAGAAAGAGTGATGAGG-3′
Tyr Rev 3 (Product size = 3496nt)	5′-GAAGTGTTAGACCAGCAGAGAC-3′
**Quikchange primers**	
Mutagenesis primer 1	5′-AGGACTCACGGTCGTCGACTCCCTTGAAGGGGAACTGAGGTCC-3′
Mutagenesis primer 2	5′-TCCGCAGTTGAAACCCATGAAGTTTCCGCTACACTGGCAGGTCCTATTATAAAAC-3′

Primer sequences for sgRNA synthesis, sequencing, and mutagenesis are shown.

Given that cleavage with WT Cas9 in the presence of a modified donor DNA was able to correct the *Tyr* defect in AB6 mice, we also examined D10A Cas9 for similar activity. We injected both guides B and D along with D10A Cas9 mRNA and the modified donor plasmid into B6 fertilized eggs (Table 1, Experiment 4). Since the fertilized eggs were derived from B6 mice, we were not able to identify successful HR via phenotype alone. We obtained 17 pups of which six were subsequently demonstrated to have indels within exon 1 of *Tyr*. While 3 of these mice had a coat color change from black to white (indicating disruption of both *Tyr* alleles), mouse 4.9 (Fig. 5, Table 2) appeared phenotypically chimeric, with both black and white patches scattered throughout. As this mouse aged, the coat color mosaicism has been maintained (discussed in more detail below). Sequence analysis identified two disrupted alleles and another allele that contained the modified donor DNA sequence (Table 3). One additional black mouse (4.4, Fig. 5b) also contained the donor insertion while maintaining the second allele without indel generation, bringing the total gene-corrected mice using both guides B and D to three out of 25 founders (12.0%). Similar frequencies for gene correction were obtained for both WT and D10A Cas9 (Table 2).

**Figure 6:**
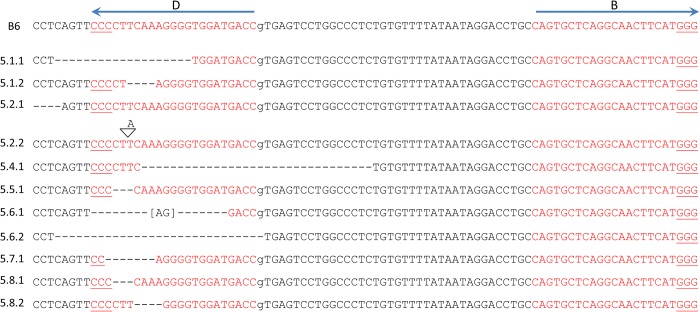
Generation of indels and donor DNA insertion following sgRNA guide D cleavage. The results of single guide D editing in AB6 mice (Experiment 5) with a donor template are depicted. Mice are numbered as in Fig. 2b. The B6 allele is indicated with the positions of guides B and D as in Fig. 1. Sequencing results from unaltered *Tyr* alleles are not shown (5.4.2, 5.5.2, 5.7.2). Deleted nt (-). Inserted nt in brackets.

Attempts to repair *Tyr* by using only one sgRNA and the modified donor DNA were not as successful as compared to when two guides were used (Table 1, Experiment 5; Table 2). Although indels were detected at expected frequencies when the modified donor was co-injected (seven of twelve or 58.3%, Table 1, Fig. 6), none of the mice exhibited donor DNA-mediated repair. Although a definitive conclusion is not possible due to small sample size, these results support the idea that co-injecting more than one sgRNA increases the likelihood of gene repair.

**Figure 7:**
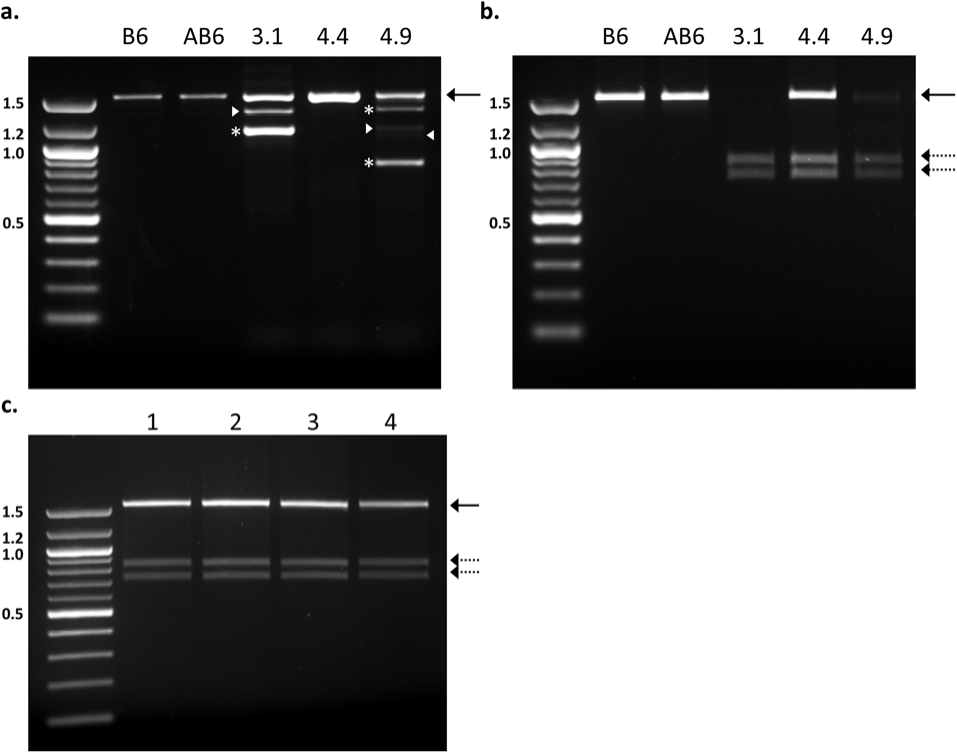
Homologous recombination (HR) in founder mice. **(a)** Genomic DNA was amplified with primers (Table 4) designed to flank the *Tyr* mutation site resulting in a PCR product of 1555bp (solid arrow). Amplification of mice 3.1 and 4.9 yielded multiple smaller molecular weight bands. The bands represented by asterisks have been confirmed by sequence analysis and represent an alternate truncated allele. Interestingly, the phenomenon of heteroduplex formation (arrowheads) is also evident in mice with more than one allele size. These artifacts are thought to occur upon heterologous binding of DNA strand from each allele (Thompson et al. 2002). Sanger sequencing of predicted heteroduplex bands confirmed the mixture of both higher and lower molecular weight bands in the product. To eliminate interference with the restriction analysis, the 1555bp band was excised from the gel for all of these mice and subjected to *Sal*I digest **(b)**. **(c)** Black-eyed progeny from mouse 4.9 all appeared to be positive for donor insertion. The predicted digestion products of 835bp and 720bp are indicated by the dashed arrow. The 100bp molecular weight marker was loaded in the left-most lane of each agarose gel with sizes as indicated (in Kb). B6: C57BL/6, AB6: C57BL/6 Albino. Mouse numbers are consistent with the text.

Finally, by engineering a *Sal*I restriction enzyme site into the donor DNA, we were able to quickly confirm donor incorporation independent of sequencing. (Ultimately, all mice were sequenced for verification.) Following PCR of the *Tyr* locus, the restriction fragment length polymorphism (RFLP) pattern provided a quick assessment of donor DNA insertion, even in the case of mosaicism (Fig. 7). Interestingly, we identified an additional allele migrating consistent with a WT band in mouse 4.9 that was resistant to *Sal*I cleavage (Fig. 7b). This allele was present in sufficiently low frequency to have been missed by our initial screening strategy but has implications on the generation of mosaicism (see below).

As summarized in Table 2, we found that the total number of mice with successful HR-mediated gene repair events was three out of 25 pups or 12.0%. All gene-repaired mice used the modified donor DNA. However, as the efficiency of targeting is also intrinsically dependent on the efficiency of Cas9 mediated DNA breaks, when expressed as a frequency of pups with detectable indels, correct donor insertion was seen three out of eleven times or 27.3% (Table 2). To summarize the influence of donor DNA on Cas9 cleavage efficiency, we found that none of the 27 pups born following co-injection with an unmodified WT donor contained any indels (Table 1, Experiment 5; Table 2). In contrast, when we expanded our analysis to all of our attempts in 3 separate experiments (Table 1, Experiments 3, 4, and 5), we found an indel frequency of 47% (18/37) when a modified donor was used (Table 2).

### Mosaicism following Cas9-mediated genome editing

Our coat color screen provides an estimate of the relative frequency of mosaicism and was later validated by offspring analysis. Indeed, our first indication that mosaicism was a possible outcome in our CRISPR-Cas9 micro-injections was the patchy coat color in one of the previously mentioned targeted mice (4.9, Fig. 5a). While sequencing analysis confirmed the presence of donor DNA in this founder, two additional deletion alleles were also identified (Fig. 5b).

We hypothesized that mosaicism in a founder mouse should lead to a non-Mendelian segregation of predicted alleles in the offspring and have demonstrated this in a number of analyzed progeny (Table 3). In mouse 3.3 (originally from AB6 host), non-Mendelian allele segregation suggested the presence of mosaicism and prompted a more careful analysis of the founder and offspring DNA; five total alleles were discovered (Table 3). Furthermore, it appears that one allele deleted at the guide D site (Δ213–224) was the substrate for cleavage by guide B at a later point (Δ213–224; Δ279–93) without loss of the intervening sequence. The pups generated from an AB6 cross to mosaic mouse 4.9 demonstrated non-Mendelian segregation of donor-containing offspring (four of five) and only one of the other two founder alleles. There is also likely a WT allele that was missed by our sequencing strategy but implied to be present during our subsequent RFLP analysis (Fig. 7b). Finally, the offspring of mouse 4.8 unexpectedly demonstrated only two of the four possible founder alleles in an apparent Mendelian segregation pattern, though more mice will need to be tested. By contrast, analysis of offspring from founder mice without apparent mosaicism demonstrates the expected Mendelian segregation ratio in multiple experiments (mice 2.3, 2.4, 2.5, 2.6, 3.1, 3.2, 5.1, 5.2, and 5.3) (Tables 1, 3).

In summary, of all founder mice with identified indels that have survived and aged sufficiently to examine coat color (n = 18), only one displayed phenotypic mosaicism (11 mice with identified indels did not survive to allow definitive phenotypic analysis of mosaicism). Two additional mice (3.3 and 4.8) harbor only genotypic mosaicism (Tables 1, 3). Therefore, even though relatively rare in our studies (three of 29 founder mice with identified indels, 10.3%, Table 2), genetic mosaicism can expand the pool of possible indels from a single founder mouse and at the same time serves as a caution against using founder CRISPR-Cas9 mice for experimental analysis without first fixing a particular allele by traditional breeding.

### Large indel generation

**Figure 8:**
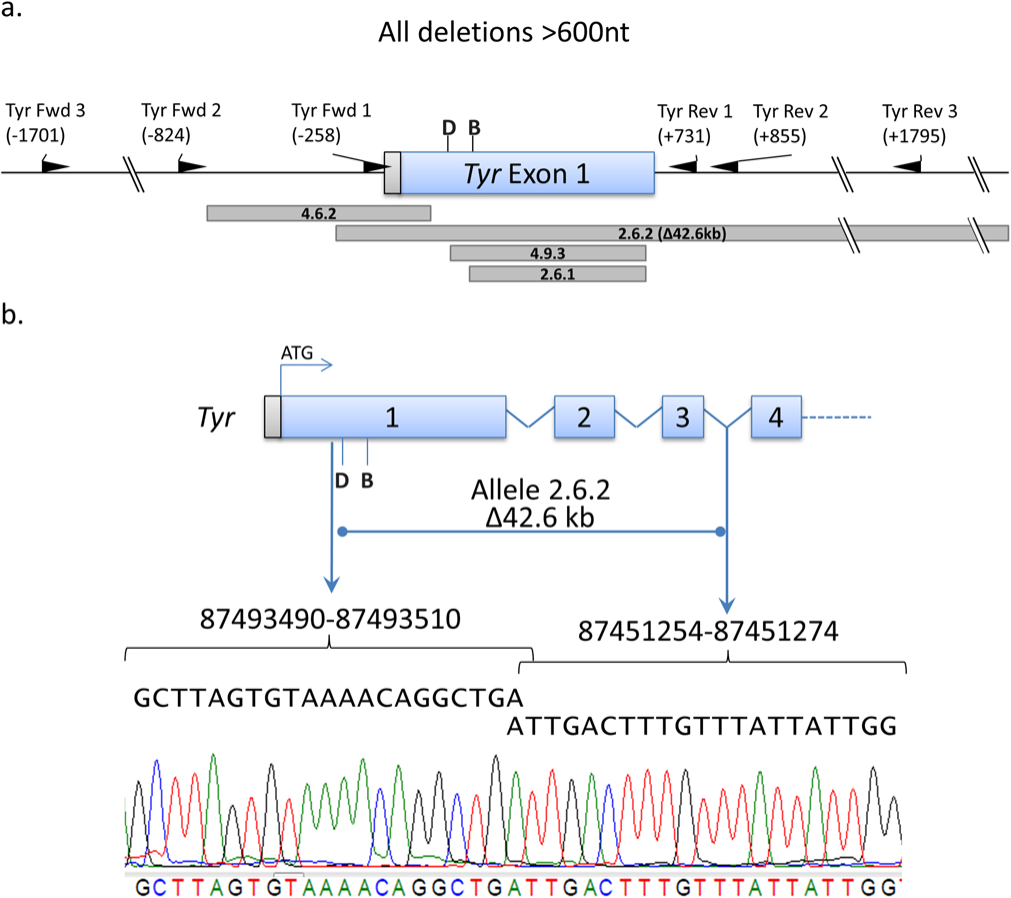
Large deletions occur with CRISPR-Cas9-mediated genome editing. **(a)** Three sets of PCR primers (Table 4) were used to identify large deletions (>600 nt) across the targeted region. The position of the 5′ end of the primer relative to predicted Cas9 cleavage site for the closest sgRNA is shown in parenthesis. The hash marks indicate regions that are not depicted in this panel. Guide B and D target sites are labeled (B) and (D), respectively. The positions of deleted nt are shown with respect the alleles in which they were identified below the schematic of the gene. **(b)** The identity of the large 42.6kb deletion from allele 2.6.2 is depicted as the sequence tracing of PCR product of the fused gene demonstrates the NHEJ repair junction. The numbering is based on the GRCm38.p2 reference assembly (mm10) for *Mus musculus*.

Our phenotypic analysis revealed the potential for large insertions or deletions (>600 nt) in the targeted genomic site that could be missed by reliance only on genotypic analysis with PCR of shorter amplicons. An example of this occurred with mouse 2.6 in which only one targeted allele was ever amplified with PCR primers *Tyr* Fwd 2 and *Tyr* Rev 2 (Table 4), located-824 nt and +855 nt relative to the Cas9 cleavage site for guide D (Fig. 8a). Since mouse 2.6 is a fully albino mouse that resulted from B6 targeting, the other *Tyr* allele must also have been targeted. Moreover, all of the pups from this cross contained either the previously identified disrupted allele (2.6.1) or the suspected large deletion allele with expected Mendelian segregation (Table 3). We reasoned that sequences for one or both of our primers for PCR amplification were within the deleted region. In order to specifically map the loss, we crossed mouse 2.6 to the A/J mouse strain and analyzed one generation of pups. A comparison of both B6 and A/J genomes performed on data generated by the Wellcome Trust Sanger Institute [34] demonstrated that the A/J strain harbors numerous SNPs in the *Tyr* locus, and we could therefore easily correlate the absence of heterogeneity at these sites with genetic loss. We ultimately determined that the large deletion was due to a loss of 42,616 nt, as confirmed by sequencing of the PCR amplicon spanning the fusion allele (Fig. 8b). We believe this is the largest deletion reported to date with the CRISPR-Cas9 genome editing system.

Similarly, we found five other mice (1.2, 4.6, 4.7, 4.8, and 4.9) either have or likely harbor large deletions (Fig. 8a). Mouse 4.6 had a 816 nt loss, and mouse 4.9 harbored a 714 nt deletion, whereas the other founder mice and/or their offspring have had only one allele sequenced. For example, sequence analysis of mouse 1.2 revealed a large insertion on one allele, and lack of a PCR product for the other allele, providing indirect evidence for an indel of at least 800 nt on either end of the Cas9 cleavage site, affecting the nearest PCR primer binding site (Fig. 8a). Analysis of the offspring from mouse 4.8 suggested inheritance of a large deletion allele in six of thirteen pups (Table 3). Specifically, while seven pups contained the 4.8.1 allele (Fig. 5b, Table 3) from the 4.8 founder and the AB6 allele from the breeding partner, the remaining six pups only demonstrated AB6 DNA sequence. Given that 4.8 was initially a B6 mouse that demonstrated a white coat color after Cas9 editing, it follows that both B6 alleles had to be disrupted for the resulting phenotype. Since in six of the pups, the only sequenced amplicons were from the AB6 breeding partner, we concluded that our PCR primers were not able to amplify the large deletion, as illustrated in several other examples above in which the large deletions were definitively identified. Therefore, the phenotypic screen revealed the presence of mutant alleles that would have otherwise gone unnoticed.

Given the total number of mice with indels in our studies (Table 1, n = 29), the incidence of a large insertion or deletion (>600 nt) is approximately 20% (Table 2), highlighting the possibility of such events being missed in PCR-based screening of short amplicons. Because of the inherent difficulty of identifying these deletions in heterozygous mice, these numbers may in fact be an underestimate. Thus, screening approaches of Cas9-modified mice should account for this possibility.

### Cas9 cleavage sites

**Figure 9:**
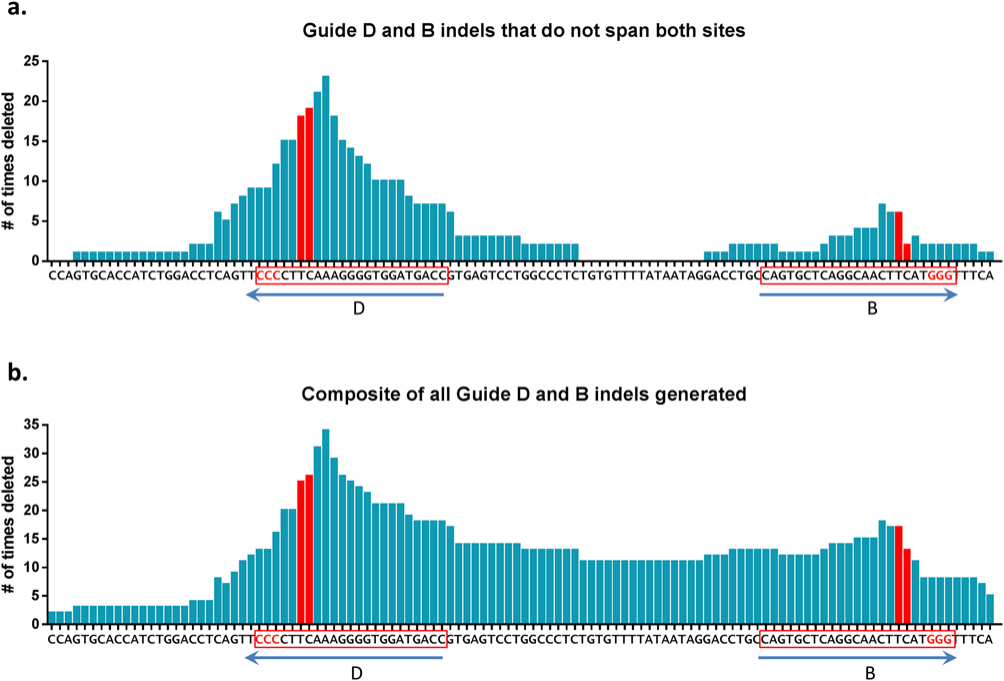
Clustered distribution of deleted nucleotides by CRISPR-Cas9-mediated genome editing. The number of times a particular nt was found to be deleted upon sequence analysis is represented by the bar above the specific nt. The DNA sequence indicates the PAM sites in red with the sgRNA recognition site for guides B and D in a box, with the guide identity indicated by the arrow below it. For each guide, the predicted cleavage site of Cas9 is indicated as the site between the two red bars. **(a)** An analysis of all deleted regions from sequence alleles containing one or two disrupted sites without the loss of the intervening residues. **(b)** As in **(a)**, but with all alleles (including intervening deletions) plotted. The data represent sequence results of 47 alleles from the 29 genome edited mice with indels.

Our studies enable a detailed and unbiased analysis of Cas9-mediated editing at the nt level. In particular, the specificity of Cas9-mediated DNA cleavage can be determined by consolidating our sequencing results across multiple experiments. Upon analysis of all of the deleted nts from all alleles where one guide cleavage site did not include the other, we found a Gaussian distribution of deleted nts peaking between positions-3 and-6, relative to the PAM site (Fig. 9a). Interestingly, Cas9 endonuclease activity from *in vitro* experiments predicts cleavage at positions -3 to -4 [35].

Upon analysis of the entire cohort of mice with identified deletions, including mice derived from injection of both guide D and B with intervening sequence loss, a similar deletion pattern emerges that is skewed further 5′ of the predicted Cas9 cleavage site (Fig. 9b). With only a smaller sampling of mice micro-injected with guide B alone, we are limited in describing its effect on deletions, though the data appear to show a similar Gaussian distribution of deleted nucleotides at positions -3 to -6 relative to the PAM site for guide B. Nonetheless, our global assessment indicates the specificity of Cas9-mediated cleavage by our guides appears to give rise to genomic modifications somewhat broader than expected from previously reported results [35].

### Reproductive success and normal life-span of targeted mice

**Figure 10:**
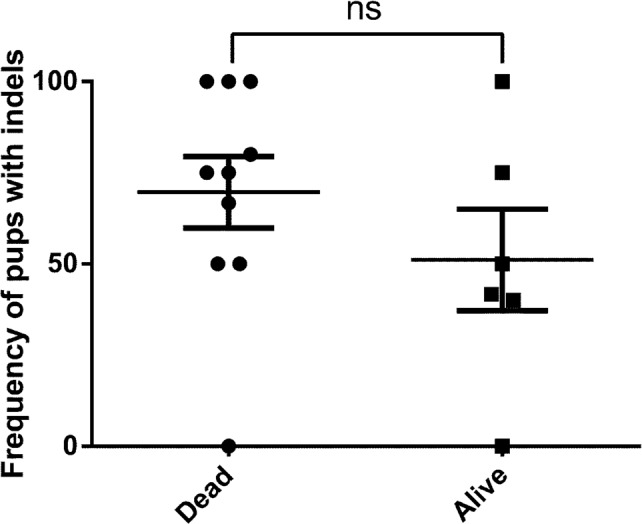
Indel frequency in Cas9-modified mice does not correlate with survival. The number of pups with indels, regardless of survival, is plotted as a frequency per injection. All mice that did not survive to at least 3 weeks were categorized as “dead”. A total of 16 independent injections (dead = 10, alive = 6) with range, mean, and SEM are plotted. *p* = 0.28, Two-tailed Student’s *t*-test. ns: non-significant.

Across multiple experiments and days of micro-injection, we observed toxicity manifested by a number of pups being either still-born or unable to survive the neonatal period that is commensurate with prior reports [23, 25]. Although it is currently unclear as to the reasons for this apparent toxicity, upon examination of the mice derived from 16 independent micro-injections, we found a relatively similar range of indel frequency among mice that survived the neonatal period and those that did not (Fig. 10; *p* = 0.28, two-tailed student’s t-test). While we did not study mice that were not born, these results indicate that survival of pups beyond the neonatal period did not correlate with the likelihood of successful Cas9-mediated genome editing at the target site.

On the other hand, representative mice from the experiments described above have been used to generate offspring to segregate the individual alleles (Table 3). With the potential for confounding off-target effects, it is crucial that one can isolate an individual allele from the founder mouse and, with conventional breeding strategies, eventually generate a homozygous line through back-crossing. This strategy requires that the founders survive to breeding age, are fertile and that the alleles are germline transmissible. Many of our targeted mice are now over twelve months old, have generated several litters, and transmitted their alleles in expected Mendelian ratios (Table 3), with the noted exceptions in the mosaic mice as described above. In the successfully targeted mice, we did not observe a gender bias or any obvious developmental, tumorigenic or fertility issues.

### Off-target identification and analysis

Given that genomic regions of close homology may exist for any sgRNA used in Cas9-dependent cleavage, consideration should be given to the possibility that these off-target sites also have been modified. In practice, we propose genetic methods to exclude off-target effects when assessing Cas9-modified mice, including breeding, analyzing multiple founders, and utilizing independent sgRNA designs. However, to directly assess the potential for off-target effects in our founder mice described here, we took advantage of the known limitations of Cas9-cleavage to guide our analysis. First, in the absence of the highly specific PAM signature (NGG), cleavage is not expected to occur, regardless of sgRNA recognition sequence homology [35]. Second, four or more mismatches between the 20nt sgRNA recognition sequence (excluding the PAM which does not provide specificity) and potential target inhibit Cas9 activity at that site [35]. Third, more than one mismatch in the 3′ half of the sgRNA recognition sequence (known as the seed sequence) is also highly unlikely to allow for DNA cleavage [36]. These three criteria formed the basis for our off-target search for Guides B and D.

Initially, we performed a BLAST search of the mouse genome with the 23nt sgRNA, iteratively replacing the “N” in the NGG motif with each alternative nt to identify targets with the highest homology. The results of this search yielded no off-target sites that met the criteria described above. Next, we used http://www.crispr.mit to search for potential off-target sites [37]. For Guide B, we identified B-OT1, B-OT2, B-OT3 and B-OT5 while for Guide D, D-OT1 and D-OT2 were identified (S1 Table). For completeness, a third search algorithm (http://gt-scan.braembl.org.au/) was used to identify potential off-target sites [38], yielding the remaining sites (B-OT4, B-OT6, B-OT7, D-OT3, and D-OT4) that fulfilled our criteria (S1 Table, S1 Fig.).

Although it is unclear why both algorithms and BLAST analysis did not identify the same sites, we screened all identified potential off-target sites for cleavage in our mice. DNA was available from 80 founder mice but in 27 mice, an unmodified donor was co-injected which resulted in inhibition of Cas9 activity on *Tyr* so these mice were excluded from analysis since it highly likely that any off target site cleavage would also be mitigated. The remaining 53 mice were grouped according to the type of Cas9 injected. All 32 mice obtained following WT Cas9 micro-injection were subjected to off-target analysis of the sites predicted in S1 Table. Since D10A Cas9 is less likely to generate genomic off-target cleavage [31, 32], we limited our analysis of mice generated from D10A Cas9 micro-injection to ten mice, nine with documented indels and one without an indel that did not survive. Thus, these 42 (32 with WT Cas9 and 10 with D10A Cas9) mice were studied for off target genome cleavage events for either sgRNA D or B and D, depending on the guide(s) used (S2 Table).

We performed Sanger sequencing across the regions of sgRNA homology (S1 Table) by analysis of PCR amplicons generated with a set of PCR primers (S3 Table). While specific amplicons could be generated for all other sites, it was not possible to do so for B-OT6 and B-OT7. Interestingly, each of these sites was identified with only one of the two search algorithms (see above). Additionally, the predicted PCR amplicons required to allow sequencing of the off-target recognition site were nearly identical for both B-OT6 and B-OT7. These off-target sites appear to be in low-complexity regions of host DNA, thus hindering our ability to specifically interrogate them. Last, these two off-target sites are located on the Y chromosome which was only present in half of the founders generated. However, we did not observe any gender bias in our founder mice, suggesting there were no profound Y chromosomal abnormalities. Thus, we performed 345 PCR and corresponding Sanger sequencing reactions to analyze the remaining potential off-target sites for 42 founder mice.

Unlike with amplification and sequencing of the *Tyr* locus at the guide recognition sites, we did not observe loss of homozygosity on the chromatogram tracing or altered mobility on DNA electrophoresis for any analyzed off-target loci. Representative chromatograms are shown from four mice which varied in their survival, type of Cas9 used, identity of indels, and in whether modified donor DNA was co-injected (S2 Fig.). By contrast, in 29 of these 42 mice, *Tyr* indels were readily identified on the tracing and/or by electrophoretic mobility. Representative tracings are shown (S2 Fig.). Finally, since it is possible that higher mortality could be observed if off-target cleavage had occurred, we assessed the survival rate in this cohort of sequenced mice. Survival, for these studies, was defined as mice that were born and remained alive beyond the initial neonatal period of seven days. Beyond this period, no additional mortality was observed in any of the founder mice. Regardless of either survival (n = 19) or death (n = 23), no animal showed any evidence of off-target cleavage (S2 Table), suggesting that we had not biased our analysis to mice based on survival beyond the neonatal period. Therefore, off-target cleavage at alternate genomic sites was not detected in our analyses.

## Discussion

Our studies provide extensive phenotypic and genetic analyses of *in vivo* Cas9-mediated genome editing of a single mouse gene. Several of our findings parallel the results in recent reports [23, 25] and importantly serve to validate our strategy. We have confirmed that Cas9-mediated DNA breaks and NHEJ repair lead to multiple distinct alleles following pronuclear injection of sgRNAs into a fertilized egg at the single cell stage. The majority of Cas9-modified founders demonstrated that mono- and bi-allelic mutations are induced with high efficiency. Our rapid phenotypic analysis provided detailed information regarding the diverse array of changes induced, and the stability and transmissibility of these genetic modifications. In addition, these studies have yielded insight into several important processes including donor repair, mosaicism, and large indel generation.

To our knowledge, this is the first report to demonstrate that donor DNA modification was required for successful HR-dependent repair induced by Cas9. Indeed, we never obtained successful repair when WT donor DNA was used. As the frequency of repair via HR is inherently low, we may not have had sufficient mice to observe the event. Alternatively, the WT donor DNA could have been cleaved after incorporation or the donor itself may have inhibited the initial CRISPR-Cas9-mediated cleavage of genomic DNA. Given the lack of indels following injection of WT donor DNA, we provide strong support for the hypothesis that genomic cleavage itself was inhibited. In this situation, the donor DNA can act as a dominant negative to prevent genome editing. Indeed, we found that the co-injection of WT donor DNA completely abolished indel formation by both guides B and D which also provides data against cleavage of incorporated donor DNA as the explanation for inhibition of gene repair. Altogether, our studies indicate that HR-mediated gene repair is prevented by simultaneous injection of WT donor DNA because of the direct inhibition of CRISPR-Cas9-mediated cleavage of genomic DNA.

In prior publications describing gene repair facilitated by CRISPR-Cas9, the sgRNA recognition site was not present in the donor DNA [21, 23, 25] and, to our knowledge, this specific requirement was not tested. In most studies, the donor DNA contained a replacement gene, such as a human orthologue of a mouse gene, or simply a knockin reporter construct having sequences flanking but not including the sgRNA recognition site. Thus, for successful HR, our findings suggest that the donor DNA needs to be modified to prevent guide RNA recognition.

Importantly, our findings that CRISPR-Cas9-mediated genome editing is inhibited by unmodified donor DNA also provides strong evidence for the specificity of cleavage. Recent reports of CRISPR-Cas9 protein-guide RNA binding to DNA *in vitro* suggest that the PAM site is first used to establish association with the target site while the nucleotides immediately 5′ of the PAM serve to increase binding affinity [39]. These studies were aided by competitor DNA fragments containing guide RNA recognition sites. Similarly, our analysis demonstrates a similar phenomenon in the fertilized egg. By supplying a donor DNA that also contains the very sequences that are being targeted, we effectively neutralized CRISPR-Cas9-mediated genome cleavage. We provide experimental evidence for CRISPR-Cas9 specificity by demonstrating that only donor DNA with alterations in the putative sgRNA recognition sites prevented genome cleavage.

Another key observation in our studies is that mosaicism can occur after CRISPR-Cas9-mediated editing. Yang *et al* initially described the occurrence of mosaicism following CRISPR-Cas9-mediated genome editing in mice [25]. They interrogated for such an event via Southern blotting and identified a few mosaics with large indels. Recently, it was shown that mosaicism can also occur without such large indels generated ([21] and this study). Mosaicism was very common in reports of genome editing employing micro-injection of DNA containing Cas9 and guide components [21, 40]. However, in our experiments mosaicism appeared to be the exception rather than the rule: phenotypic mosaicism rarely occurred and was confirmed by both sequencing and offspring analysis.

Our low rate of mosaicism using RNA components allows us to draw several conclusions regarding the timing and mechanism of CRISPR-Cas9-mediated DNA cleavage and repair in the developing embryo. Conceptually, mosaicism could be explained by an active Cas9/sgRNA complex that partitions when the zygote divides. Depending on the half-life of the editing complex and its activity, multiple alleles could be generated by independent cleavage and repair events occurring in different daughter cells at the 2-cell stage or beyond. However, since we rarely observed mosaicism, our results strongly suggest that Cas9 mRNA and sgRNA, when injected into the pronucleus of a fertilized egg, acts almost exclusively at the single cell stage for several reasons. First, the vast majority of the founder mice analyzed (with the mosaic exceptions described above) only harbored one or two alleles of the targeted *Tyr* locus. Second, the offspring from the founder mice only received one of the two alleles identified in the original founder and did so with the expected Mendelian frequencies. Finally, even though the source of founder DNA was a heterogeneous population of cells from a tail sample, it accurately represented the alleles found in the germline of that mouse as determined by genetic breeding experiments. Together, these results demonstrate that highly efficient, CRISPR-Cas9-mediated genome editing after RNA introduction via pronuclear micro-injection as described here is limited to a short period of time in the fertilized egg itself.

Implicit in the generation of mosaic mice is the presence of an allele with an unmodified sgRNA site in the genome at the 2-cell stage or beyond because CRISPR-Cas9-mediated cleavage destroys the recognition site for a given guide. Two requirements must therefore be met for mosaicism to occur. First, genome editing in the single cell stage should be incomplete so that an unaltered target site remains intact in the daughter cell. This notion suggests that mosaic animals are more likely to harbor the unaltered WT allele. Consistent with this consideration, we found a WT *Tyr* allele in two of the three mosaic mice described here. Furthermore, the potential for subsequent re-cleavage of donor DNA after incorporation (and cell division) was also associated with a high rate of mosaicism [21]. Second, there must be sufficient Cas9/sgRNA complexes to partition into subsequent generations. Indeed, Cheng *et al* demonstrated that Cas9 and sgRNA complexes retain functional activity at the four-cell stage when micro-injected into single-cell embryos [41]. In Drosophila embryos, as the concentration of injected RNA was reduced, the incidence of mosaic adults decreased dramatically, consistent with dilutional effects upon cell division [42]. These considerations suggest that with one sgRNA, two alleles are possible if Cas9 acts fully at the single-cell stage and three alleles if Cas9 acts partially at the 1-cell stage and fully at the 2-cell stage. With two sgRNAs, two alleles are possible if Cas9 acts fully at the 1-cell stage, while five alleles if Cas9 acts partially at the 1-cell stage and fully at the 2-cell stage. Therefore, inefficient CRISPR-Cas9-mediated cleavage in the single-cell stage would allow the persistence of modifiable WT alleles for subsequent cleavage, and generation of mosaicism.

In contrast to previous work, we found a high rate of large deletions in the founder mice. We detected deletions of greater than 600 nt in nearly 20% of our mice with indels. In fact, in one mouse we observed a very large deletion of greater than 42 kb. We believe the identification of these large deletions, generated by both WT and D10A Cas9, was aided by our phenotypic screen and offspring analysis. While other studies have also described CRISPR-Cas9-modified mice with large deletions (up to 2.2 kb), all of these studies seem to be limited to injections of sgRNA pairs, rather than individual sgRNAs [27, 43, 44]. Similarly, large deletions appear to be favored when paired sgRNAs were used in our study, suggesting that the mechanism for such large indel generation is dependent on two simultaneous DNA breaks. Importantly, the possibility of large deletions underscores the limitation of screens based on analysis of short PCR products. Two potential mechanisms for large indel generation have been proposed: MMEJ and SSA [13, 15]. While they share some of the same features, the molecules involved are not all the same. It is not clear which of these particular mechanisms, if either, is induced following CRISPR-Cas9-mediated DSB breaks, especially in the case of our identified large 42 kb deletion. Genomic context, sgRNA sequence, and/or Cas9 may play a yet unrecognized role in promoting large-scale DNA end resection.

Our analysis of the deleted nucleotides differs somewhat from studies that previously established the site of CRISPR-Cas9-mediated cleavage. It is not clear if this broader range of cleaved nucleotides is sequence-dependent, a phenomenon of *in vivo* activity, or a reflection primarily of the repair process. Notably, the original report describing the identification of the Cas9 cleavage site was performed in a cell-free system without the influence of NHEJ repair [35]. Our observed Gaussian distribution of nucleotide loss within 10 nt of the cleavage site is consistent with an initial cleavage at the Cas9 site followed by classical NHEJ repair, similar to what has been described *in vitro* with iPS and tumor cell lines [6]. In the current report, the results also indicate that the deletion event favors nucleotides upstream rather than downstream of the PAM. Interestingly, the mechanism of NHEJ may dictate the degree of deletion observed. For example, plants have been shown to primarily harbor 1 nt indels after CRISPR-Cas9-mediated editing [45], and may reflect differences in NHEJ as compared to mammals.

We have demonstrated that the majority of alleles identified in the founder mice are transmitted to the progeny, regardless of whether the founder was an identified mosaic. This demonstrates that the somatic mutations we identified in the tail DNA arose from the genetic changes induced in the single-cell embryo and was subsequently maintained with high fidelity in the germ cells. We also observed a high but reproducible level of toxicity following RNA micro-injection, commensurate with prior reports [23, 25]. One explanation for these findings is that CRISPR-Cas9-mediated cleavage might result in large chromosomal translocations that would be deleterious to newborn mice. Alternatively, disruption of an unidentified off-target site may promote the mortality observed. While we did not observe any statistical differences in the detection of indels in mice that survived to adulthood compared with mice that were either still-born or died in the neonatal period, we cannot rule out the possibility of larger unidentified chromosomal changes or off-target effects. Nonetheless, we were able to breed many of our *Tyr* mutant mice which did not have any obvious defects, suggesting that these mice had limited off-target effects, though we may be underestimating these effects in non-viable pups as mentioned.

We interrogated potential off-target cleavage events by selecting candidate off-target regions for amplification and sequencing of 42 mice, including those in which *Tyr* indels had occurred. Although web tools such as those used here have given researchers a starting point [37, 38], it is important to emphasize that sequencing of predicted off-target sites is essentially a test of the available algorithms. Even upon analysis of the sites most likely to be susceptible to Cas9-mediated cleavage by our guide RNAs, we discovered no off-target cleavage sites in mice targeted by the sgRNA described in this report to disrupt *Tyr*. This finding is consistent with similar reports in the literature [25, 46]. The absence of off-target effects following micro-injection of fertilized eggs as compared with their more frequent occurrence *in vitro* [47, 48] may indicate that higher target specificity is achieved in zygotes, though different guides and targets were studied [49]. One limitation of our studies is that low level-mosaicism at the off target sites would not be evident in the sequence tracings but our studies would still indicate that off target cleavage is still relatively rare if it occurs at all. Although another approach to analyze off-target cleavage would be deep sequencing of the entire genome of the founder mice, this also has technical limitations. For example, even unmodified mice can display genetic changes but these could be due to chance or the inherent error-rate of the sequencing technology [50]. Finally, our analysis of off-target effects did not include mice that were not born. Some of these mice could have died from deleterious effects of Cas9-mediated cleavage though many other factors affect the successful birth of micro-injected zygotes. Nevertheless, our inability to detect off-target genomic indels in pups that were born is likely due to our thorough analyses prior to micro-injection whereby potential sgRNAs with demonstrated high homology (by BLAST analysis as described) to off-target sites were not used.

The capacity of CRISPR-Cas9-mediated genome editing to generate multiple different alleles from one sgRNA injection will allow assignment of specific phenotypes to individual modified genes. Each newly targeted mouse will have one or more unique alleles in terms of genetic modification by NHEJ and if a null mutation is produced, it should give rise to the same phenotype as other mice with homozygous null mutations in the same gene. Independently, use of alternate sgRNAs for the same gene generates another cohort of targeted mice that should also have the same phenotype. Since these alleles can be passed in the germline, as demonstrated here, the newly generated phenotype(s) should also track with the mutation in their offspring. The offspring can subsequently be intercrossed to achieve a novel homozygous strain for phenotypic analysis. On the other hand, each sgRNA has the potential for distinct off-target effects that will not track with the targeted gene of interest in offspring analysis. Thus, CRISPR-Cas9-mediated off-target effects should be avoidable while simultaneously providing several founder mouse lines with different mutations for verification thereby adding an advantage to conventional gene targeting of ES cells where usually only one targeted mouse is studied.

An additional benefit of generating multiple alleles of a targeted gene is the capacity to interrogate amino acid deletions in terms of function. Several of our mutated mice contained in-frame deletions. In one of these mice, a deletion of 5 amino acids resulted in an active tyrosinase molecule as determined by eye color, indicating that these residues are not required for appropriate gene function. Thus, CRISPR-Cas9-mediated genome editing could inform study of proteins by alteration of functional domains.

Our findings have obvious implications for gene corrected stem cells for therapies in humans. Specifically, for homozygous recessive disorders, it is theoretically possible to isolate particular stem-cell subsets from a patient, correct the gene defect, and clone the corrected cells for expansion and reintroduction to the patient. For this technique to be feasible, one would need to efficiently generate CRISPR-Cas9-mediated cleavage initially followed by extinguishing the DNA cleavage activity of Cas9 upon cell division to avoid continued Cas9 activity, in addition to minimizing any off-target effects of the guide RNAs used. For patients with heterozygous deleterious genes manifesting dominant negative effects, a guide RNA selective for only the deleterious allele would be ideal.

In summary, we have extensively analyzed both genotypic and phenotypic data following CRISPR-Cas9-mediated genome editing of a single mouse gene. Our studies reveal several critical parameters in greater detail. These results provide the foundation to interpret and predict the diverse outcomes following such genome-editing experiments that will become more commonplace as the field continues to advance.

## Materials and Methods

### Mice

C57BL/6 (C57BL/6Tac) and ICR/CD1 mice were purchased from Taconic (Hudson, NY) and AB6 mice were purchased from The Jackson Laboratory (Bar Harbor, ME).

### sgRNA design

While NCBI BLAST (http://blast.ncbi.nlm.nih.gov/) was initially used to assess sequence similarity of potential sgRNAs, various online tools have since become available to facilitate the process of sgRNA design and selection. These include E-CRISP (http://www.e-crisp.org/E-CRISP/)[51], the *CRISPR Design Tool* from the Zhang lab (http://crispr.mit.edu/)[37], and GT-Scan (http://gt-scan.braembl.org.au/gt-scan/)[38]. Upon further analysis, these tools confirmed the correct and specific targeting our sgRNA designs. The sequences of guide RNAs are shown in Table 4.

### RNA synthesis

A T7 polymerase initiation site was added to the 5-prime end of the sgRNA (Table 4) as described previously [23]. We found that although PAGE purification of the long forward primer is recommended to increase yield, sufficient quantities of sgRNA can be synthesized without this costly and time-consuming step. All primers were synthesized by IDT. We used the pX330 (Addgene plasmid #42230 [32]) template for both sgRNA and Cas9 synthesis. For sgRNA synthesis a PCR product was generated that included a T7 polymerase transcription initiation sequence followed by the 20 nt guide RNA sequence (not including the 3 nt PAM), and the tracRNA sequence from plasmid pX330 [5, 52]. For Cas9-D10A transcription, modified primers and the pX335 (Addgene plasmid #42335 [5]) template were employed (Table 1). PCR amplification was performed with HF-Phusion DNA polymerase (New England Biolabs (NEB), Beverly, MA) and the product was purified through a spin column (for sgRNA; PCR purification kit, Clontech Laboratories, Mountain View, CA) or by organic extraction and ethanol precipitation (for Cas9). After purification, an aliquot was checked for quality by gel electrophoresis and quantified on a Nanodrop 1000 (ThermoFisher Scientific, Waltham, MA). RNA synthesis was performed according to manufacturer (Life Technologies, Carlsbad, CA) recommendations (MEGAshortscript T7 for sgRNA and mMESSAGE mMACHINE T7 Ultra for Cas9 mRNA). While the sgRNA was purified through a spin column (MEGAclear RNA purification kit (Life Technologies), the Cas9 mRNA was less prone to degradation when precipitated with lithium chloride according to the alternate protocol described by the manufacturer. sgRNA and Cas9 mRNA were diluted in nuclease-free injection buffer, aliquoted into single use tubes, and frozen at -80°C. Individual tubes were later thawed and assessed for integrity via the 2200 TapeStation Instrument (Agilent Technologies, Santa Clara, CA).

### Donor DNA construction

Donor DNA was created by PCR amplification (HF-Phusion, NEB) of the host gene and TOPO subcloning (Life Technologies). Homology arms were generated to encompass 800 nt on either side of the intended substitutions using the primers *Tyr* Fwd 2 and *Tyr* Rev 2 (Table 4). The sgRNA recognition sites were modified with Quickchange (Agilent Technologies) to contain alternate codons that still maintained the translated protein sequence.

### Micro-injection

C57BL/6NTac female mice 4 weeks old were super-ovulated and mated with C57BL/6NTac males. Day 0.5 single cell embryos were isolated and underwent pronuclear micro-injection in the Department of Pathology Micro-injection Core using standard methods [53]. The embryos were co-injected with sgRNA at 10–20 ng/µl, Cas9 at 100–200 ng/µl, and plasmid donor at 10 ng/µl in DNase/RNase free micro-injection buffer, 1 mM Tris, 0.25 mM EDTA pH 7.4. This results in a four-fold molar excess of sgRNA to Cas9 mRNA. 20–25 injected embryos were transferred into the oviducts of d0.5 pseudopregnant ICR/CD1 recipient female mice. Albino B6 mice were used as breeders. One day of injection refers to an independent reconstitution of RNA and/or DNA reagents, multiple rounds of micro-injection and subsequent implantation into several pseudo-pregnant recipients limited to a single day.

### DNA methods

Genomic DNA was prepared from tail tissue of founder mice and their offspring using the Puregene extraction kit (Qiagen, Venlo, Netherlands) following manufacturer recommendations. PCR amplification using 100 ng of genomic DNA was performed with HF-Phusion DNA polymerase (NEB) and the *Tyr* primers (Table 4). The PCR products were then directly sequenced or cloned and sequenced with BigDye v3.1 per manufacturer recommendations (Life Technologies). For establishing donor insertion, the same *Tyr* primers used to generate the 1555 nt product (Table 4) were used for amplification as above, followed by gel purification (Gel Extraction Kit, Clontech). The purified PCR product was then subjected to *Sal*I (NEB) digest for 30 min at 37°C followed by electrophoresis on a 1.5% agarose gel and visualized with ethidium bromide staining. A 100 bp or 1 kb ladder (NEB) was also applied for molecular weight determination.

### Off-target analysis

Genomic DNA was prepared from tail tissue of founder mice as using the Puregene extraction kit (Qiagen). PCR amplification using 100ng of genomic DNA was performed with HF-Phusion DNA polymerase (NEB) and the off-target primers listed in S3 Table as well as *Tyr* using the primes listed in Table 4. The off-target loci were chosen as described in the text. PCR products were then directly sequenced with BigDye v3.1 per manufacturer recommendations (Life Technologies). Sequence chromatograms were viewed and analyzed for loss of homozygosity using FinchTV v1.4.0 (PerkinElmer, Waltham, MA).

### Ethics statement

This study was carried out in strict accordance with the recommendations in the Guide for the Care and Use of Laboratory Animals of the National Institutes of Health. The protocol was approved by the Animal Studies Committee at Washington University School of Medicine under animal protocol 20130049A2.

## Supporting Information

S1 Fig Potential off-target cleavage sites following CRISPR-Cas9-mediated genome editing. The sequences of sgRNA guides B **(a)** and D **(b)** are aligned above the off-target sites identified as described in the text. The off-target sites are depicted with ten additional nt flanking the region of homology. The PAM is in blue, the high specificity seed sequence is in red and the rest of the homologous region is shown in blue. Each nt that diverges from the sgRNA sequence is indicated by a shaded box. Off-target site names are shown to the right of their respective sequences.
(TIF)

S2 Fig Representative sequencing chromatograms across *Tyr* and the potential off-target cleavage sites. Four founder mice (S2 Table) are depicted here (numbered to the left of the tracings) and are representative of the lack of off-target events seen in all 42 mice subjected to an identical analysis. Indels were only generated at the intended on-target *Tyr* locus. The sequence of the off-target site is indicated above the first tracing in each group. The arrow head is closest to the PAM. An arrow pointing to the left indicates that the complementary strand was sequenced. An arrow pointing to the right indicates that the non-complementary strand was sequenced. For the off-target sites, the region of homology to the respective guide is indicated by the shaded area. *Tyr* tracings show examples of how we detected indels in *Tyr* as evident by heterozygous tracings. Loss of homozygosity (LOH) is indicated by the downward arrow. The deletions previously described for mice 2.2, 2.4, and 2.9 correspond to these heterozygous regions. Sequencing these regions results in LOH if the alternate allele is WT (mouse 2.4) or has a second deletion nearby (mice 2.2 and 2.9). Mouse 4.8 harbors a nine nt deletion (as indicated under the panel) that was preferentially amplified and sequenced; the corresponding region in the predominant alternate allele has a deletion in this region (Allele 4, Table 3). The guide binding sites have been lost or shortened as indicated in the shaded region.
(TIF)

S1 Table Off-target sites for the sgRNAs used in this study. (DOC)

S2 Table Characteristics of founder mice analyzed for off-target cleavage. (DOC)

S3 Table Off-target primer sequences. (DOC)
