# Supplementary figures and images for: Detailed Phenotypic and Molecular Analyses of Genetically Modified Mice Generated by CRISPR-Cas9-Mediated Editing
Source: PLoS One. 2015 Jan 14;10(1):e0116484. doi: 10.1371/journal.pone.0116484 (PMC4294663; doi:10.1371/journal.pone.0116484)

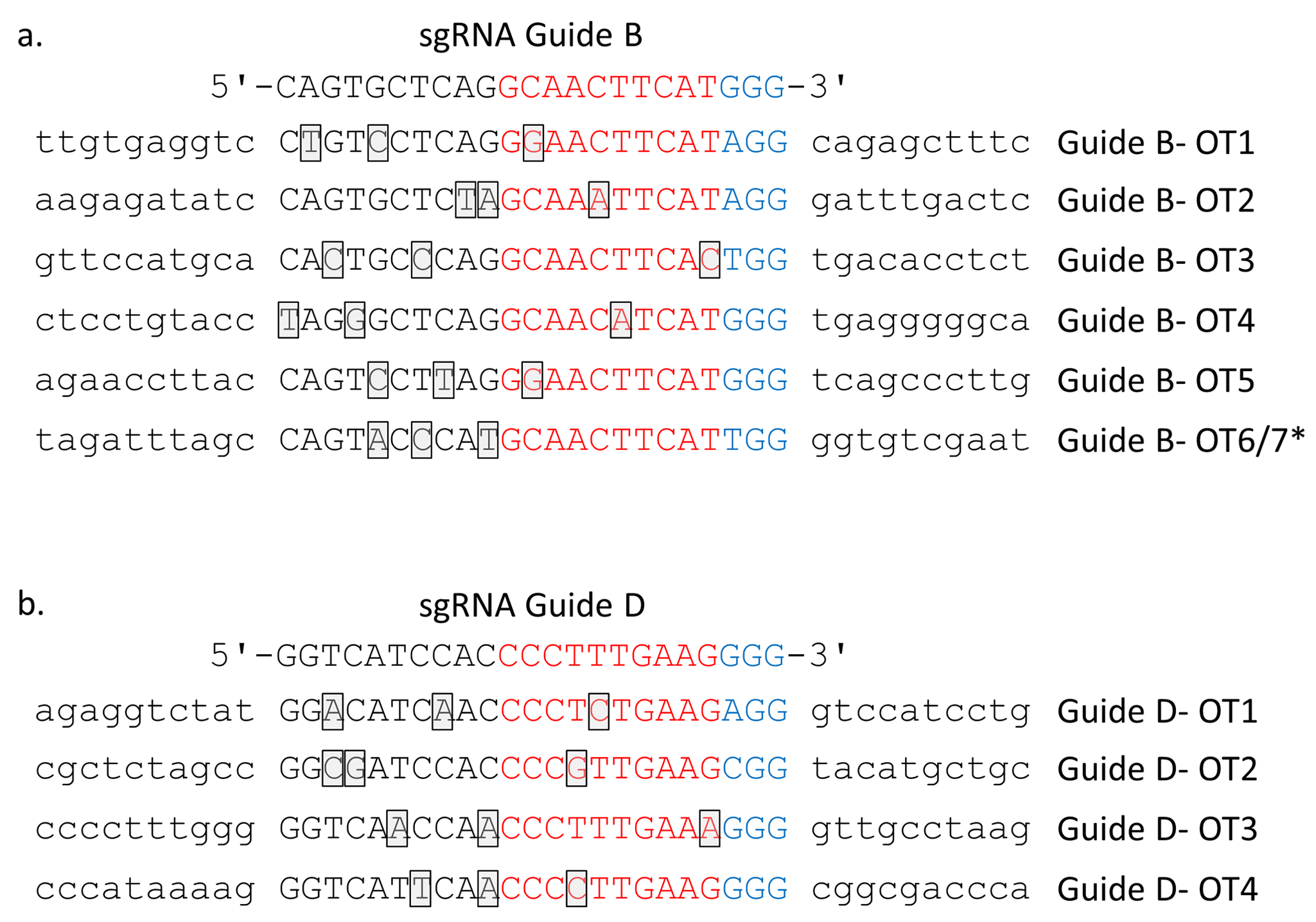

Supplement: S1 Fig — The sequences of sgRNA guides B (a) and D (b) are aligned above the off-target sites identified as described in the text. The off-target sites are depicted with ten additional nt flanking the region of homology. The PAM is in blue, the high specificity seed sequence is in red and the rest of the homologous region is shown in blue. Each nt that diverges from the sgRNA sequence is indicated by a shaded box. Off-target site names are shown to the right of their respective sequences. (TIF) [file pone.0116484.s001.tif]

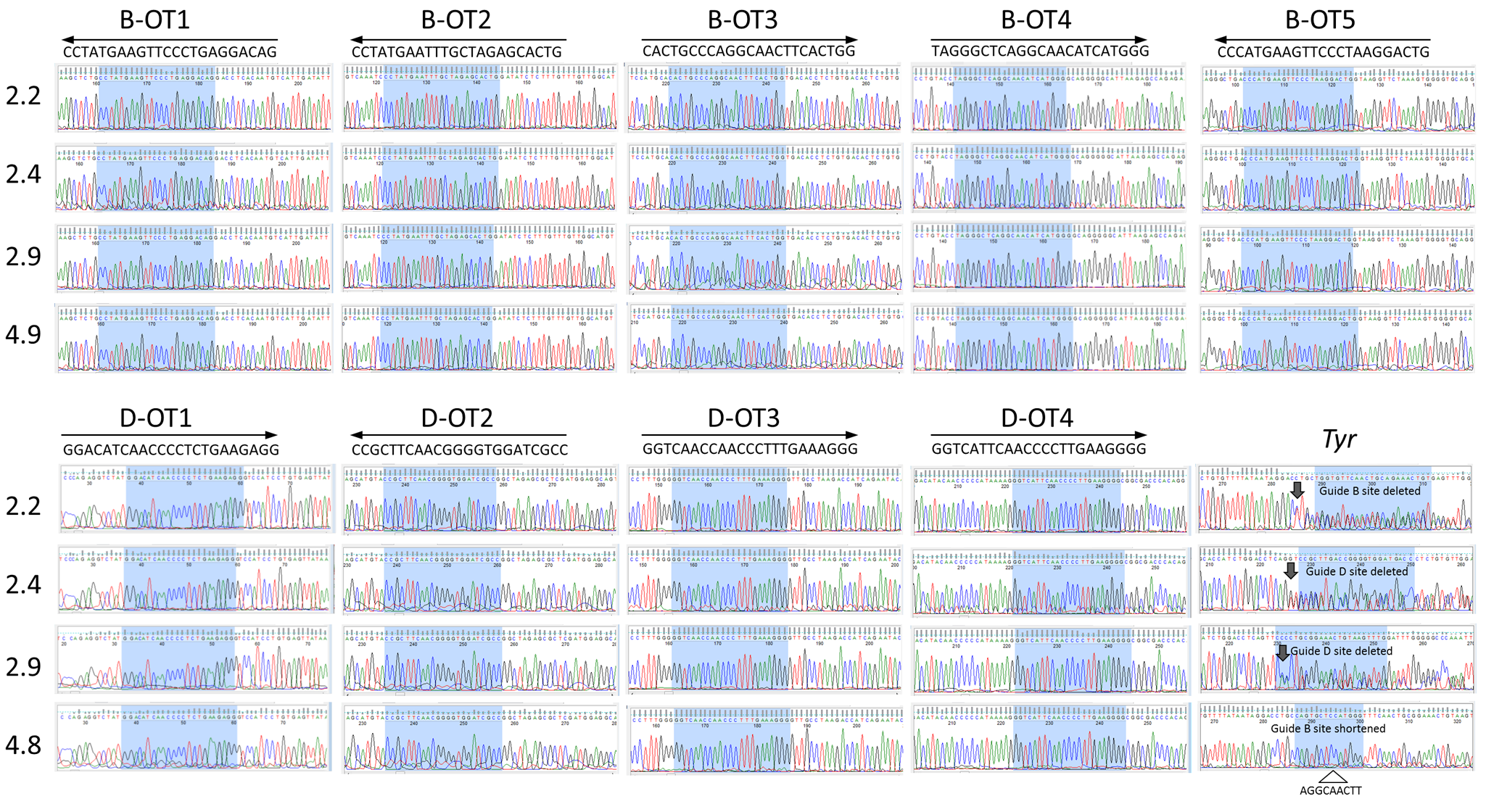

Supplement: S2 Fig — Four founder mice (S2 Table) are depicted here (numbered to the left of the tracings) and are representative of the lack of off-target events seen in all 42 mice subjected to an identical analysis. Indels were only generated at the intended on-target Tyr locus. The sequence of the off-target site is indicated above the first tracing in each group. The arrow head is closest to the PAM. An arrow pointing to the left indicates that the complementary strand was sequenced. An arrow pointing to the right indicates that the non-complementary strand was sequenced. For the off-target sites, the region of homology to the respective guide is indicated by the shaded area. Tyr tracings show examples of how we detected indels in Tyr as evident by heterozygous tracings. Loss of homozygosity (LOH) is indicated by the downward arrow. The deletions previously described for mice 2.2, 2.4, and 2.9 correspond to these heterozygous regions. Sequencing these regions results in LOH if the alternate allele is WT (mouse 2.4) or has a second deletion nearby (mice 2.2 and 2.9). Mouse 4.8 harbors a nine nt deletion (as indicated under the panel) that was preferentially amplified and sequenced; the corresponding region in the predominant alternate allele has a deletion in this region (Allele 4, Table 3). The guide binding sites have been lost or shortened as indicated in the shaded region. (TIF) [file pone.0116484.s002.tif]
