# Supplementary material for: Detailed Phenotypic and Molecular Analyses of Genetically Modified Mice Generated by CRISPR-Cas9-Mediated Editing
Source: PLoS One. 2015 Jan 14;10(1):e0116484. doi: 10.1371/journal.pone.0116484 (PMC4294663; doi:10.1371/journal.pone.0116484)
Supplement: S1 Table — (DOC) [file pone.0116484.s003.doc]

**Table S1: Off-target sites for the sgRNAs used in this study**

| **Potential Off-target Sequence**  Guide B:  CAGTGCTCAGGCAACTTCATGGG | Number of Mismatches | | | Chr | Position | Str | Name | Gene |  |
| --- | --- | --- | --- | --- | --- | --- | --- | --- | --- |
|  | Total | **H** | **L** |  |  |  |  |  |  |
| C**T**GT**C**CTCAGG**G**AACTTCATAGG | 3 | 1 | 2 | 13 | 75859955 | - | B-OT1 | no |  |
| CAGTGCTC**TA**GCAA**A**TTCATAGG | 3 | 1 | 2 | 2 | 149754478 | - | B-OT2 | *Gm14133* lincRNA |  |
| CA**C**TGC**C**CAGGCAACTTCA**C**TGG | 3 | 1 | 2 | 2 | 6210460 | + | B-OT3 | *Echdc3* Intron |  |
| **T**AG**G**GCTCAGGCAAC**A**TCATGGG | 3 | 1 | 2 | 14 | 74500402 | - | B-OT4 | no |  |
| CAGT**C**CT**T**AGG**G**AACTTCATGGG | 3 | 1 | 2 | 10 | 76824945 | - | B-OT5 | *Pcbp3* Intron |  |
| CAGT**A**C**C**CA**T**GCAACTTCATTGG | 3 | 0 | 3 | Y | 12432796 | + | B-OT6 | no |  |
| CAGT**A**C**C**CA**T**GCAACTTCATTGG | 3 | 0 | 3 | Y | 17144695 | + | B-OT7 | no |  |
|  | | | | | | | | |  |
| **Potential Off-target Sequence**  Guide D:  GGTCATCCACCCCTTTGAAGGGG | Number of Mismatches | | | Chr | Position | Str | Name | Gene |  |
|  | Total | **H** | **L** |  |  |  |  |  |  |
| GG**A**CATC**A**ACCCCT**C**TGAAGAGG | 3 | 1 | 2 | 5 | 30637677 | + | D-OT1 | no |  |
| GG**CG**ATCCACCCC**G**TTGAAGCGG | 3 | 1 | 2 | 14 | 79183038 | - | D-OT2 | *Vwa8* Exon |  |
| GGTCA**A**CCA**A**CCCTTTGAA**A**GGG | 3 | 1 | 2 | 8 | 124923666 | + | D-OT3 | *Egln1* Intron |  |
| GGTCAT**T**CA**A**CCC**C**TTGAAGGGG | 3 | 1 | 2 | 8 | 113857924 | + | D-OT4 | no |  |

A list of potential off-target sites are shown, along with the number and position of mismatches, the target chromosome (Chr), the position of the cleavage site (based on numbering from the GRCm38.p2 reference assembly (mm10) for *Mus musculus*), the strand of DNA the target site is located on (str), the name of the off-target site, and if a genetic element was located at the site. Total numbers of mismatches are indicated as well as the number of mismatches within the high (H) specificity seed sequence (orange), or lower (L) specificity upstream region (blue). The "N" of the NGG PAM is indicated in green.
