# Supplementary material for: Detailed Phenotypic and Molecular Analyses of Genetically Modified Mice Generated by CRISPR-Cas9-Mediated Editing
Source: PLoS One. 2015 Jan 14;10(1):e0116484. doi: 10.1371/journal.pone.0116484 (PMC4294663; doi:10.1371/journal.pone.0116484)
Supplement: S2 Table — (DOC) [file pone.0116484.s004.doc]

**Table S2: Characteristics of founder mice analyzed for off-target cleavage.**

| **Mouse** | **Cas9** | **sgRNA** | **Donor** | **Indel** | **Survival** |
| --- | --- | --- | --- | --- | --- |
| 1.1 | WT | D | none | Yes | Dead |
| 1.2 | WT | D | none | Yes | Dead |
| 1.3 | WT | D | none | Yes | Dead |
| 1.4 | WT | D | none | No | Dead |
| 1.5 | WT | D | none | Yes | Dead |
| 2.1 | D10A | B+D | none | No | Dead |
| 2.2* | D10A | B+D | none | Yes | Dead |
| 2.3 | WT | B+D | none | No | Alive |
| 2.4* | WT | B+D | none | Yes | Alive |
| 2.5 | WT | B+D | none | Yes | Alive |
| 2.6 | WT | B+D | none | Yes | Alive |
| 2.7 | WT | B+D | none | No | Dead |
| 2.8 | WT | B+D | none | Yes | Dead |
| 2.9* | WT | B+D | none | Yes | Dead |
| 2.10 | WT | B+D | none | Yes | Dead |
| 3.1 | WT | B+D | Mod | Yes | Alive |
| 3.2 | WT | B+D | Mod | Yes | Alive |
| 3.3 | WT | B+D | Mod | Yes | Alive |
| 3.4 | WT | B+D | Mod | No | Alive |
| 3.5 | WT | B+D | Mod | No | Alive |
| 3.6 | WT | B+D | Mod | No | Alive |
| 3.12 | WT | B+D | Mod | No | Dead |
| 3.18 | D10A | B+D | Mod | Yes | Dead |
| 3.19 | D10A | B+D | Mod | Yes | Dead |
| 4.1 | D10A | B+D | Mod | Yes | Alive |
| 4.4 | D10A | B+D | Mod | Yes | Alive |
| 4.6 | D10A | B+D | Mod | Yes | Alive |
| 4.7 | D10A | B+D | Mod | Yes | Alive |
| 4.8* | D10A | B+D | Mod | Yes | Alive |
| 4.9 | D10A | B+D | Mod | Yes | Alive |
| 5.1 | WT | D | Mod | Yes | Alive |
| 5.2 | WT | D | Mod | Yes | Alive |
| 5.4 | WT | D | Mod | Yes | Dead |
| 5.5 | WT | D | Mod | Yes | Dead |
| 5.6 | WT | D | Mod | Yes | Dead |
| 5.7 | WT | D | Mod | Yes | Dead |
| 5.8 | WT | D | Mod | Yes | Dead |
| 5.9 | WT | D | Mod | No | Dead |
| 5.10 | WT | D | Mod | No | Alive |
| 5.11 | WT | D | Mod | No | Dead |
| 5.12 | WT | D | Mod | No | Dead |
| 5.13 | WT | D | Mod | No | Dead |

These 42 founder mice were subjected to off-target analysis. Mice indicated with an (*) are representative of the remaining mice and the sequence chromatograms for the off-target sites are shown in Figure S2. Cas9 (WT or D10A nickase); sgRNA (Guide D alone or both D and B); Donor (either no donor or the modified donor (Mod)); Indels ("yes" if an indel in *Tyr* was identified); Survival (still-born pups and those not surviving beyond five days of life were classified as "dead").
