# Supplementary material for: Detailed Phenotypic and Molecular Analyses of Genetically Modified Mice Generated by CRISPR-Cas9-Mediated Editing
Source: PLoS One. 2015 Jan 14;10(1):e0116484. doi: 10.1371/journal.pone.0116484 (PMC4294663; doi:10.1371/journal.pone.0116484)
Supplement: S3 Table — (DOC) [file pone.0116484.s005.doc]

**Table S3: Off-Target Primer Sequences**

| **Off-target primer name** | **Primer Sequence** |
| --- | --- |
| B-OT1-Fwd | 5'-GCACTCAGTCAGAGCACACT-3' |
| B-OT1-Rev | 5'-GTCCACCACCAGGACTTCAG-3' |
| B-OT2-Fwd | 5'-GGCAGTGACAGGCTGATTCT-3' |
| B-OT2-Rev | 5'-AGCGTAGGCAGCAACTCTTT-3' |
| B-OT3-Fwd | 5'-CCTTCCGGACTCGCTGTTAG-3' |
| B-OT3-Rev | 5'-TCTGGTTCCAGCTAGGGTGA-3' |
| B-OT4-Fwd | 5'-CAGTTACCTGTGTTACAATGACTC-3' |
| B-OT4-Rev | 5'-GCTGCTTCTGTAGGATCTAGCC-3' |
| B-OT5-Fwd | 5'-TCGGATGAGCTGTGTTTGCT-3' |
| B-OT5-Rev | 5'-CAGACACTGGGGACAAGCAT-3' |
| B-OT6/7-Fwd | 5'-TCCTGGTGACACTTCATGGC-3' |
| B-OT6/7-Rev | 5'-AGTGTCTCATCTCCGTGGGA-3' |
| D-OT1-Fwd | 5'-GTACGGGAAGACAACCCTGG-3' |
| D-OT1-Rev | 5'-AAAAGGTTGAGAGAGCCGGG-3' |
| D-OT2-Fwd | 5'-CCTCCGGACGGTAGCTTTAC-3' |
| D-OT2-Rev | 5'-GACGCTCTACCACTGGAACC-3' |
| D-OT3-Fwd | 5'-GGGTCCTGCCACCTCTAAAC-3' |
| D-OT3-Rev | 5'-CTTGGCAGAAACCCTCGTCT-3' |
| D-OT4-Fwd | 5'-CCCCACTTGCATTGGCTCTA-3' |
| D-OT4-Rev | 5'-GTACCAGCAAGGTGGGACAA-3' |
